# Structure and mechanism of *Staphylococcus aureus* oleate hydratase (OhyA)

**DOI:** 10.1074/jbc.RA120.016818

**Published:** 2021-01-09

**Authors:** Christopher D. Radka, Justin L. Batte, Matthew W. Frank, Brandon M. Young, Charles O. Rock

**Affiliations:** 1 The Department of Infectious Diseases, St Jude Children’s Research Hospital, Memphis, Tennessee, USA; 2 Department of Chemical Biology and Therapeutics, St Jude Children’s Research Hospital, Memphis, Tennessee, USA

**Keywords:** *Staphylococcus aureus* (*S. aureus*), X-ray crystallography, bacteria, enzyme catalysis, fatty acid metabolism, flavin adenine dinucleotide (FAD), oleate hydratase (OhyA), FAD, flavin adenine dinucleotide, *h*18:0, (*R*)-10-hydroxyoctadecanoic acid, OhyA, oleate hydratase, PEG400, polyethylene glycol 400

## Abstract

Flavin adenine dinucleotide (FAD)-dependent bacterial oleate hydratases (OhyAs) catalyze the addition of water to isolated fatty acid carbon–carbon double bonds. *Staphylococcus aureus* uses OhyA to counteract the host innate immune response by inactivating antimicrobial unsaturated fatty acids. Mechanistic information explaining how OhyAs catalyze regiospecific and stereospecific hydration is required to understand their biological functions and the potential for engineering new products. In this study, we deduced the catalytic mechanism of OhyA from multiple structures of *S. aureus* OhyA in binary and ternary complexes with combinations of ligands along with biochemical analyses of relevant mutants. The substrate-free state shows Arg81 is the gatekeeper that controls fatty acid entrance to the active site. FAD binding engages the catalytic loop to simultaneously rotate Glu82 into its active conformation and Arg81 out of the hydrophobic substrate tunnel, allowing the fatty acid to rotate into the active site. FAD binding also dehydrates the active site, leaving a single water molecule connected to Glu82. This active site water is a hydronium ion based on the analysis of its hydrogen bond network in the OhyA•PEG400•FAD complex. We conclude that OhyA accelerates acid-catalyzed alkene hydration by positioning the fatty acid double bond to attack the active site hydronium ion, followed by the addition of water to the transient carbocation intermediate. Structural transitions within *S. aureus* OhyA channel oleate to the active site, curl oleate around the substrate water, and stabilize the hydroxylated product to inactivate antimicrobial fatty acids.

Oleate hydratase (OhyA^3^) (EC 4.2.1.53) is a bacterial unsaturated fatty acid hydratase that catalyzes water addition to the 9-*cis* double bond in oleic acid to form (*R*)-10-hydroxyoctadecanoic acid (*h*18:0) (1, 2, 3). OhyA is widely distributed in bacteria and was first recognized as a component in the reduction of linoleic acid by commensal rumen bacteria (4). Hydroxy fatty acids impact bacterial cell surface hydrophobicity, acid and bile stress tolerance, and adherence to host cells (5, 6), and OhyA has potential commercial value for the production of hydroxy fatty acids for the food, chemical, and cosmetic industries (7). Recent developments point to important functions of OhyA in immune regulation. Hydroxy-fatty acids produced by commensal bacteria act as signaling molecules to reduce gut inflammation (8, 9), and unsaturated fatty acids inhibit bacterial growth by destabilizing membranes (10, 11, 12, 13). The pathogen *Staphylococcus aureus* is particularly sensitive to palmitoleic acid (16:1Δ9*Z*), the major antimicrobial unsaturated fatty acid produced by the innate immune system (14), and the expression of OhyA counteracts the effects of antimicrobial fatty acids on *S. aureus* membranes (15). OhyA is packaged into immunomodulatory extracellular membrane vesicles that are released from *S. aureus* during infection (16). These biochemical functions of OhyA make it a key determinant for *S. aureus* virulence in an endocarditis infection model (17) validating the importance of the enzyme in pathogenesis.

The OhyA protein family is a large group of bioinformatically related hydratases with different selectivities for unsaturated fatty acid substrates (18, 19). X-ray crystal structures reveal that OhyA family members have a conserved fold belonging to the c.3.1.2 flavin adenine dinucleotide (FAD)-linked reductases, amino-terminal domain protein family in the Structural Classification of Proteins extended database (scop.berkeley.edu) (20, 21, 22). OhyA requires FAD; however, OhyA belongs to the group of FAD-dependent enzymes where FAD does not play a redox role in the chemical mechanism (23). FAD is proposed to either play a structural role in organizing the active site (24) or to participate in catalysis by either stabilizing a positive charge or donating a proton to the double bond of an unsaturated fatty acid (20, 25). OhyAs have a consensus dinucleotide-binding motif/phosphate-binding signature sequence (26, 27, 28) and a conserved RGGR**E**M OhyA active site Loop. The *Lactobacillus acidophilus* OhyA structure identified a putative hydrophobic fatty acid binding tunnel that extends into the interior from the carboxy-terminal surface, but the tunnel is not contiguous with the active site (21). The *Elizabethkingia meningoseptica* OhyA structure captures a complex with a polyethylene glycol 400 (PEG400) molecule in the active site (20). This complex enabled identification of FAD-interacting residues and the glutamate on the OhyA active site Loop that coordinates the active site water (20). Based on the low activity of the *E. meningoseptica* OhyA(E122A) and (Y241F) mutants (20), it was proposed that the isolated double bond removes the Tyr241 hydroxyl proton simultaneous with the attack of water to produce the 10-hydroxy product. This proposed mechanism is not consistent with the established chemical mechanism for the addition of water to isolated double bonds (29).

This study defines the OhyA structural elements and conformational changes that promote alkene attack on an active site hydronium ion in an acid-catalyzed hydration mechanism. The oleate carbonyl leads the fatty acid into the OhyA substrate binding tunnel where it is prevented from entering the active site by Arg81. The conformational changes upon FAD binding simultaneously rotate Arg81 out of the tunnel allowing the fatty acid to rotate into the active site and repositions Glu82 to coordinate the only remaining water in the dehydrated active site. This water is a hydronium ion based on the hydrogen bond network observed in the OhyA•PEG400•FAD substrate analog complex consisting of Glu82, the dipole of helix α7, and the hydroxyl of PEG400. The hydronium ion is attacked by the double bond, and water adds to the carbocation generated on carbon-10. The OhyA•*h*18:0•FAD product complex is stabilized by a hydrogen bond network linking the backbone carbonyl of Phe187, the hydroxyl of *h*18:0, the tyrosyl oxygen of Tyr201, and the backbone carbonyl of Val505. Our data indicate the reaction proceeds by the classical acid-catalyzed mechanism for alkene hydration via a hydronium ion and rules out the current model for OhyA catalysis that posits the reaction is initiated by deprotonation of Tyr201 (20, 23, 30, 31, 32).

## Results

### S. aureus OhyA

**Figure 1:**
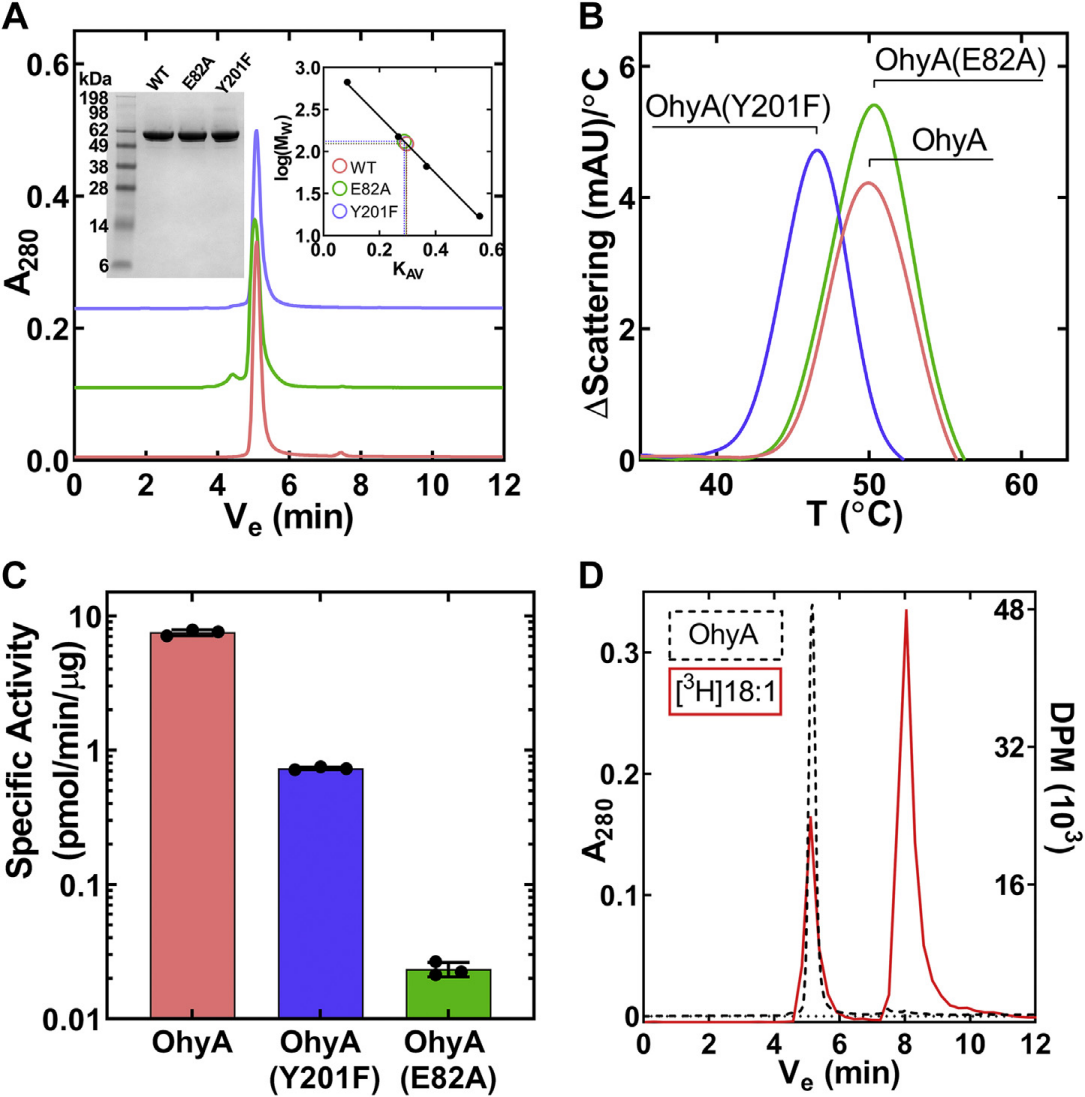
**Purification and properties of *S. aureus* OhyA and its mutant derivatives.***A*, Gel filtration chromatography of OhyA, OhyA(E82A), and OhyA(Y201F) indicates they are dimers. *Left inset*, SDS gel electrophoresis shows protein purity. *Right inset*, molecular weight calculation using calibration standards described in *Experimental procedures*. *B*, Representative first derivative plots of light scattering versus temperature for OhyA (48.2 °C ± 1.1 °C), OhyA(E82A) (49.0 °C ± 0.6 °C), and OhyA(Y201F) (45.6 °C ± 0.3 °C). Temperatures are reported as mean ± standard error (n = 3). *C*, Specific activities of OhyA (7.547 ± 0.221 pmol/min/μg), OhyA(Y201F) (0.735 ± 0.010 pmol/min/μg), and OhyA(E82A) (0.023 ± 0.03 pmol/min/μg). Specific activities are reported as mean ± standard error (n = 3). *D*, Gel filtration chromatogram shows OhyA binds [^3^H]oleate in the absence of FAD. OhyA was monitored by A_280_ (*dashed black line*) and [^3^H]oleate was monitored by scintillation counting (DPM) (*red line*). FAD, flavin adenine dinucleotide; OhyA, oleate hydratase.

OhyA and its mutant derivatives were expressed as His-tagged proteins and purified by Ni^2+^ affinity and gel filtration chromatography. Each protein was pure as judged by gel electrophoresis, and size-exclusion chromatography indicates that OhyA and its mutant derivatives are dimers in solution (Fig. 1*A*). Glu82 and Tyr201 are two residues predicted to inactivate OhyA based on the current model for catalysis (20, 23, 30, 31, 32). UV light scattering upon thermal denaturation was used to probe the impact of the OhyA(E82A) and OhyA(Y201F) mutations on protein stability. The thermal transition temperatures (T_agg_) for ligand-free OhyA and OhyA(E82A) were the same, but OhyA(Y201F) was 3 °C less stable indicating that the Tyr201 hydroxyl participates in an interaction that stabilizes OhyA (Fig. 1*B*). The two mutant proteins are not equally inactive as reported by Engleder *et al*. (20). The specific activity of OhyA(Y201F) was ∼10-fold lower than OhyA, and OhyA(E82A) was a >100-fold less active (Fig. 1*C*). An OhyA•oleate complex is isolated by gel filtration chromatography in the absence of FAD (Fig. 1*D*), showing that OhyA is a fatty acid binding protein, and oleate binds independently of FAD like other flavoprotein-dependent enzymes (33).

### OhyA functional domains

**Figure 2:**
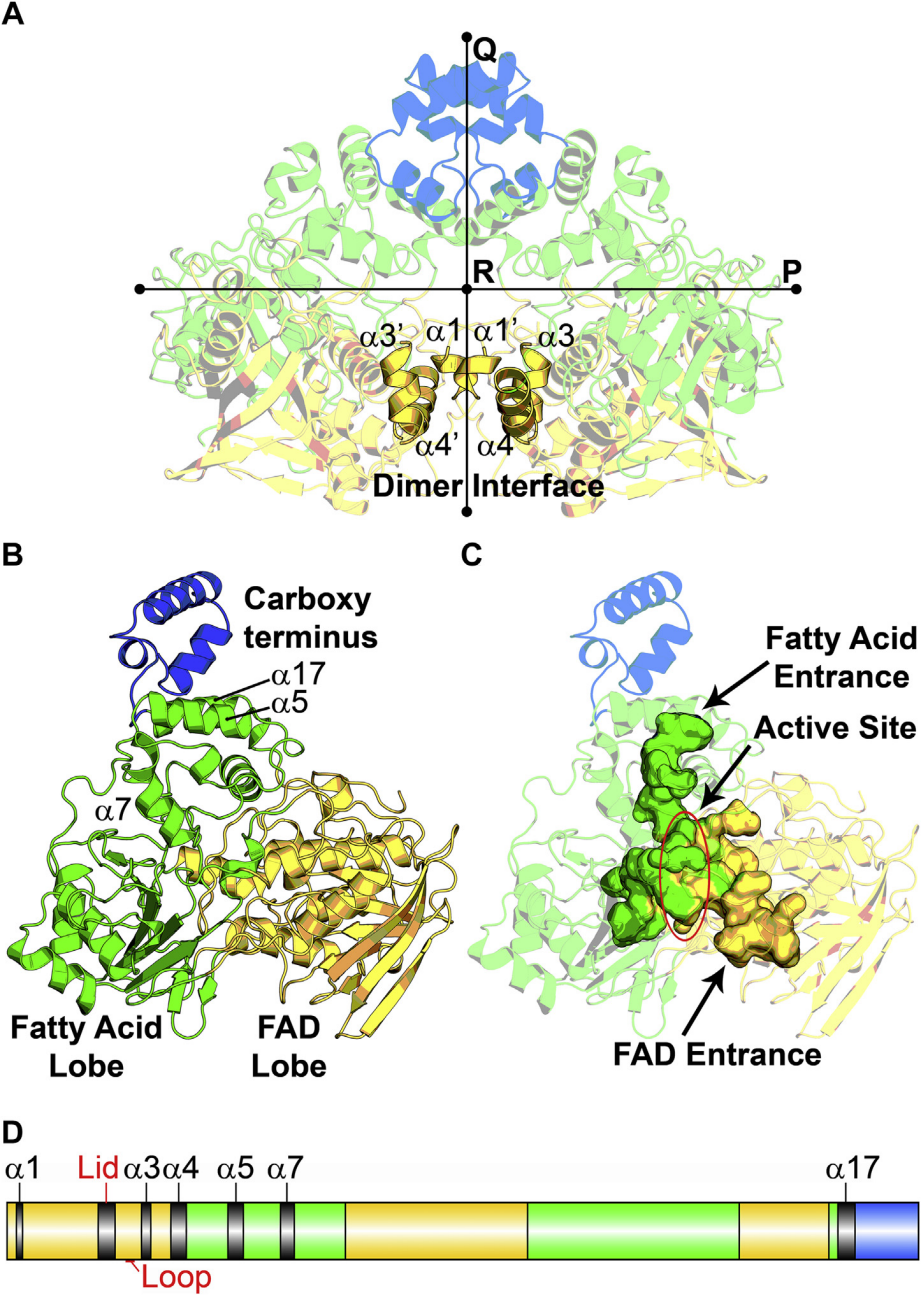
**OhyA is a dimer composed of three functional domains.** The fatty acid lobes are *green*, FAD lobes are *yellow*, and carboxy termini are *blue*. *A*, OhyA proteins use interacting helices α1, α3, and α4 to form the OhyA dimer interface along the Q axis. *B*, The OhyA protomer is divided into three functional domains: FAD lobe, fatty acid lobe, and carboxy terminus. *C*, The interior cavity of an OhyA protomer is mapped in solid color showing the contributions of the fatty acid (*green*) and FAD (*yellow*) lobes. Fatty acids enter the hydrophobic tunnel just below the carboxy terminus and FAD enters through the FAD lid at the opposite end. The active site is located at the intersection of the fatty acid and FAD cavities. *D*, The functional domains are mapped onto the primary sequence. The locations of key α helices, the sequence the covers FAD (Lid), and the active site loop (Loop) are indicated. FAD, flavin adenine dinucleotide; OhyA, oleate hydratase.

*S. aureus* OhyA is a prototypical member of Family 11 in the fatty acid hydratase database (18). OhyA crystallizes with three protomers in the asymmetric unit (Fig. S1*A*), and the X-ray data collection and refinement statistics for all structures are listed in Table 1. Two protomers interact with each other to form the dimer, and the third protomer interacts with a symmetry-related protomer around a crystallographic 2-fold axis to form a dimer with a protomer in the adjacent asymmetric unit. OhyA dimerizes along the 2-fold noncrystallographic Q symmetry axis (Fig. 2*A*). Each protomer has a surface area of ∼25,000 Å^2^, and ∼2500 Å^2^ (10%) is primarily buried by the interacting helices α1, α3, and α4 in the amino terminal region that form the dimerization interface (Fig. 2*A*). All OhyA proteins are dimers (15, 20, 21, 22), except for the *Rhodococcus erythropolis* OhyA ortholog, which is a monomeric enzyme that lacks the key α1 dimerization helix (34).

**Table 1:** Data collection and model refinement statistics

Parameter	OhyA•PEG400	OhyA•PEG400•FAD	OhyA(E82A)	OhyA(E82A)•18:1	OhyA(E82A)•18:1•FAD
PDB	7KAV	7KAW	7KAX	7KAY	7KAZ
Data collection					
Space group	C121	C121	C121	C121	C121
Cell dimensions					
a, b, c (Å)	188.8, 113.2, 118.8	188.1, 112.9, 119.1	189.7, 113.9, 119.8	189.0, 113.5, 119.3	188.0, 113.1, 118.4
α, β, γ (°)	90, 117.2, 90	90, 117.0, 90	90, 117.2, 90	90, 117.0, 90	90, 116.9, 90
Resolution (Å)	50–1.84 (1.87–1.84)	23–2.10 (2.17–2.10)	30–3.51 (3.56–3.51)	30–1.95 (1.98–1.95)	22–1.85 (1.90–1.85)
Unique reflections	175,248 (17,688)	126,745 (12,711)	27,502 (2483)	150,514 (10,700)	183,821 (18,078)
R_pim_	0.046 (0.196)	0.023 (0.135)	0.072 (0.170)	0.041 (0.399)	0.040 (0.485)
Redundancy	7.1 (6.8)	7.3 (7.1)	3.8 (3.2)	3.5 (3.1)	3.8 (3.8)
Completeness (%)	91.9 (94.6)	98.9 (99.4)	97.7 (94.6)	97.7 (97.1)	98.5 (96.3)
I/σ(I)	14.0 (2.7)	7.8 (2.3)	8.4 (4.5)	14.0 (2.8)	6.4 (2.0)
Wilson B factor	21.0	28.8	55.3	20.3	19.4
Refinement					
Refinement resolution (Å)	47–1.84 (1.91–1.84)^a^	23–2.10 (2.18–2.10)	30–3.51 (3.64–3.51)	29–1.95 (2.02–1.95)	22–1.85 (1.92–1.85)
Reflections used in refinement	174,994 (17,669)	126,645 (12,709)	27,497 (2483)	150,486 (10,700)	183,776 (18,078)
Reflections used for R _free_	1998 (205)	2004 (204)	2007 (181)	2002 (145)	2005 (205)
R_work_ (%)	0.1704 (0.2200)	0.1975 (0.2943)	0.1864 (0.2264)	0.1611 (0.2028)	0.1995 (0.3660)
R_free_ (%)^b^	0.1933 (0.2460)	0.2345 (0.3280)	0.2477 (0.2929)	0.1875 (0.2485)	0.2525 (0.4215)
No. of nonhydrogen atoms	15,831	16,095	13,921	16,326	16,992
Macromolecules	14,132	14,167	13,912	14,085	14,099
Ligands	83	187	0	74	168
Solvent	1616	1741	9	2167	2725
Ramachandran statistics (%)					
Favored	97.40	96.28	92.80	97.41	96.14
Allowed	2.42	3.43	6.91	2.30	3.75
Outliers	0.17	0.29	0.29	0.29	0.12
Root-mean-square deviations					
Bond lengths (Å)	0.007	0.008	0.004	0.007	0.013
Bond angles (°)	1.11	1.19	0.95	1.14	1.46
Clashscore	6.47	8.02	8.09	6.29	6.28
Average B factor	26.3	35.9	49.9	24.8	27.7
Macromolecules	25.2	35.0	49.9	22.8	25.7
Ligands	34.6	37.0	-	30.2	32.9
Solvent	35.6	42.8	31.7	38.0	37.7

FAD, flavin adenine dinucleotide; OhyA, oleate hydratase; PBD, Protein Data Bank; PEG400, polyethylene glycol 400.

a Statistics for the highest-resolution shell are shown in parentheses.

b R_free_ test set uses ∼5% of the data.

The *S. aureus* OhyA protomer has a bilobed structure for binding two substrates and a peripheral carboxy-terminal helical domain with an overall structure that is the same as reported for other OhyAs (Fig. 2*B*). Volkov *et al*. (21) divides the protein into four structural elements (I-IV; Fig. S1*B*); however, here we use a functional classification to describe the OhyA protomer domains (Fig. 2*C*). The FAD lobe contains the conserved dinucleotide binding motif/phosphate (FAD) binding signature sequence, the active site Loop, and the Lid covering the FAD entrance (Fig. S2). The FAD-binding domain (Fig. 2*B*) is a variant of the Rossmann fold consisting of a parallel five-stranded β-sheet flanked by three α-helices on one side and an additional crossover connection consisting of an antiparallel three-stranded β-sheet and α-helix on the other side (Fig. S1*C*). This topology is also observed in the FAD-containing glutathione reductase family (35) and corresponds to structural element I (Fig. S1*C*). The fatty acid lobe (Fig. 2*B*) creates a hydrophobic tunnel with an entrance just below the carboxy terminal domain that extends to the active site (Fig. 2*C*) and is constructed from structural elements II and III (Fig. S1*D*). The internal cavity in the FAD lobe merges with the fatty acid tunnel to form the active site at the domain interface (Fig. 2*C*). The carboxy terminal domain consists of three α-helices (Fig. 2*B*) and constitutes the structural element IV (Fig. S1*B*). The amphipathic helices have hydrophobic side chains facing inward to form a hydrophobic surface cavity and the polar side chains extend into the solvent. Abundant lysine and arginine residues give this region a positively charged electrostatic surface. The OhyA lobes are encoded in a nonlinear fashion using crossover connections to piece the lobe elements together to form the functional domain (Fig. 2*D*). The amino terminal element of the FAD lobe contains the dimerization helices (α1, α3, and α4), the sequence of 12 amino acids that covers the FAD binding site (Lid), and the conserved RGGR**E**M active site Loop (Fig. 2*D*). The fatty acid lobe encodes helices α5 and α17 that form the entrance to the substrate tunnel, and helix α7 contributes the α-helix dipole to the active site. These highly conserved structural features do not change in our crystal structures, except for the Lid and the Loop sequences which undergo significant conformational changes depending on the bound ligands.

### Fatty acid binding

**Figure 3:**
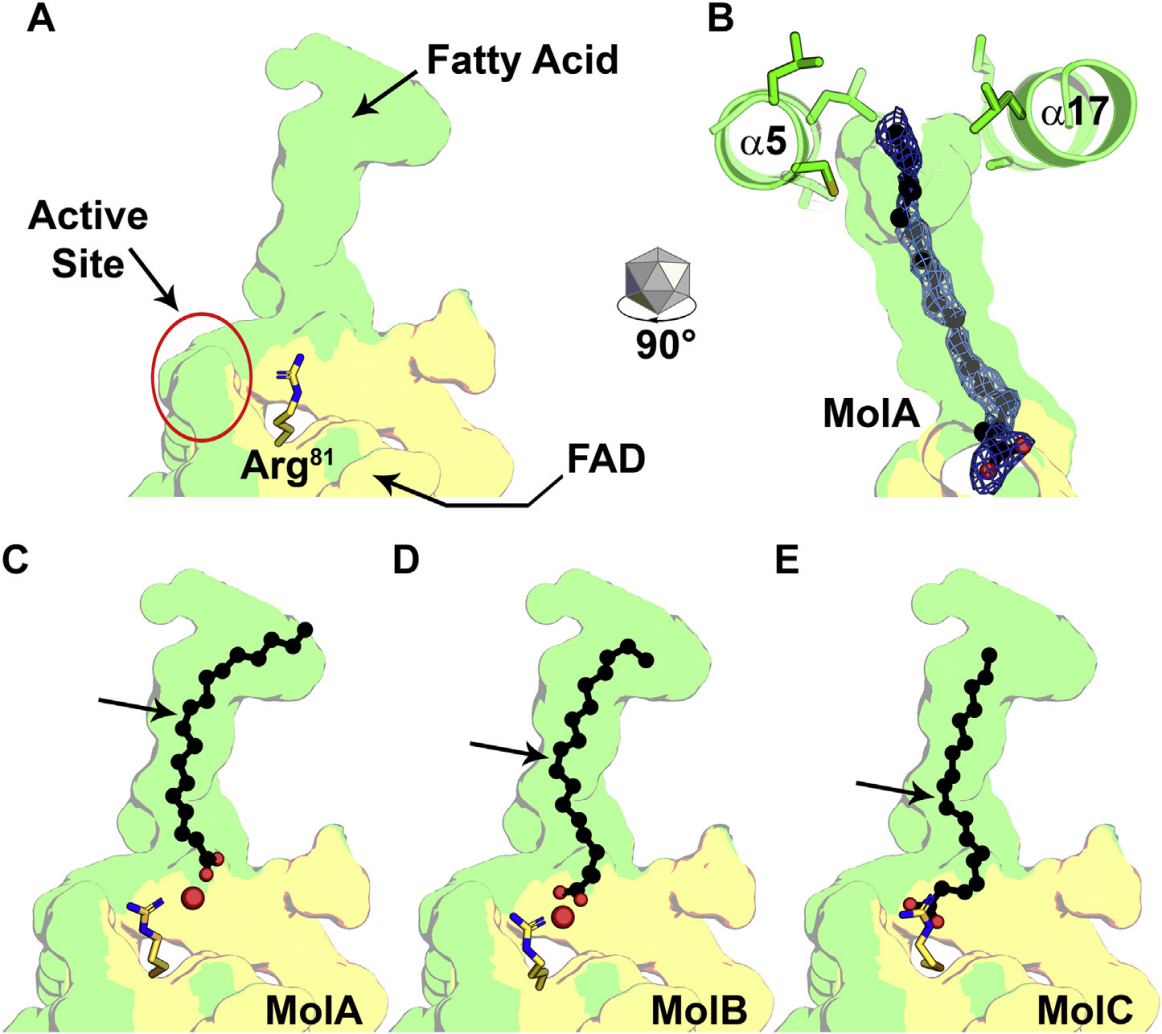
**OhyA is a fatty acid binding protein.** The transparent interior cavity surfaces are formed by the FAD (*yellow*) and fatty acid (*green*) lobes. *A*, View of the empty tunnel in the OhyA(E82A) structure (PDB: 7KAX). The Arg81 side chain walls off the end of the substrate binding tunnel at the entrance to the active site (*red circle*). *B*, The mouth of the tunnel is created by hydrophobic residues on helices α5 and α17. Oleate (*black*) sits in the substrate binding tunnel with its tail at the tunnel mouth in OhyA(E82A)•oleate complex (PDB: 7KAY, MolA). The oleate electron density calculated from a 2mF_O_ − DF_C_ map contoured at 1 σ (blue mesh) shows the carboxyl group pointing toward the bottom of the tunnel. *C*, The OhyA(E82A)•oleate complex (PDB: 7KAY, MolA) shows the fatty acid tail at the tunnel entrance and a water molecule (red sphere) between oleate and Arg81. *D*, In OhyA(E82A)•oleate complex (PDB: 7KAY, MolB) oleate is further into the tunnel forming an oleate–water–Arg81 hydrogen bond network. *E*, The water is absent from the tunnel, and oleate makes direct hydrogen bond contacts with Arg81 in OhyA(E82A)•oleate complex (PDB: 7KAY, MolC). *Arrows* denote the positions of the Δ9 double bond. FAD, flavin adenine dinucleotide; OhyA, oleate hydratase.

Glu82 in the active site Loop (RGGR**E**M) is critical for activity (Fig. 1*C*), and we mutated this residue to slow catalysis and capture complexes that were not obtained with the wild-type enzyme. The OhyA(E82A) structure in the absence of ligands (Protein Data Bank [PDB]: 7KAX) reveals the state of the empty fatty acid tunnel (Fig. 3*A*). The catalytic Loop residue Arg81 stands upright to create a wall at the end of the tunnel preventing the fatty acid from entering the active site. The OhyA(E82A)•oleate complex structure was obtained by soaking OhyA(E82A) crystals with oleate delivered with bovine serum albumin (PDB: 7KAY). Helices α5 and α17 form the tunnel entrance that is lined with hydrophobic amino acids that guide oleate into the tunnel (Fig. 3*B*). The complete electron density for oleate shows the fatty acid tail at the tunnel entrance with the carboxyl group leading the fatty acid toward the active site (Fig. 3*B*). Each of the protomers in the asymmetric unit of OhyA(E82A)•oleate complex (PDB: 7KAY) shows oleate in a slightly different location as it advances down the binding tunnel (Fig. S3). In MolA, there is a water molecule between the fatty acid carboxylate and Arg81, and the fatty acid tail extends to the tunnel entrance (Fig. 3*C*). In MolB, the fatty acid carboxylate moves closer to Arg81 forming an oleate–water–Arg81 hydrogen bond network (Fig. 3*D*). In MolC, the intervening water is absent, and the carboxylate forms direct hydrogen bond interactions with Arg81 (Fig. 3*E*). Oleate is blocked from entering the active site by the sidechain of Arg81. These data establish the location of the substrate binding tunnel in OhyA and the orientation of oleate as it approaches the active site.

### OhyA•FAD interactions

**Figure 4:**
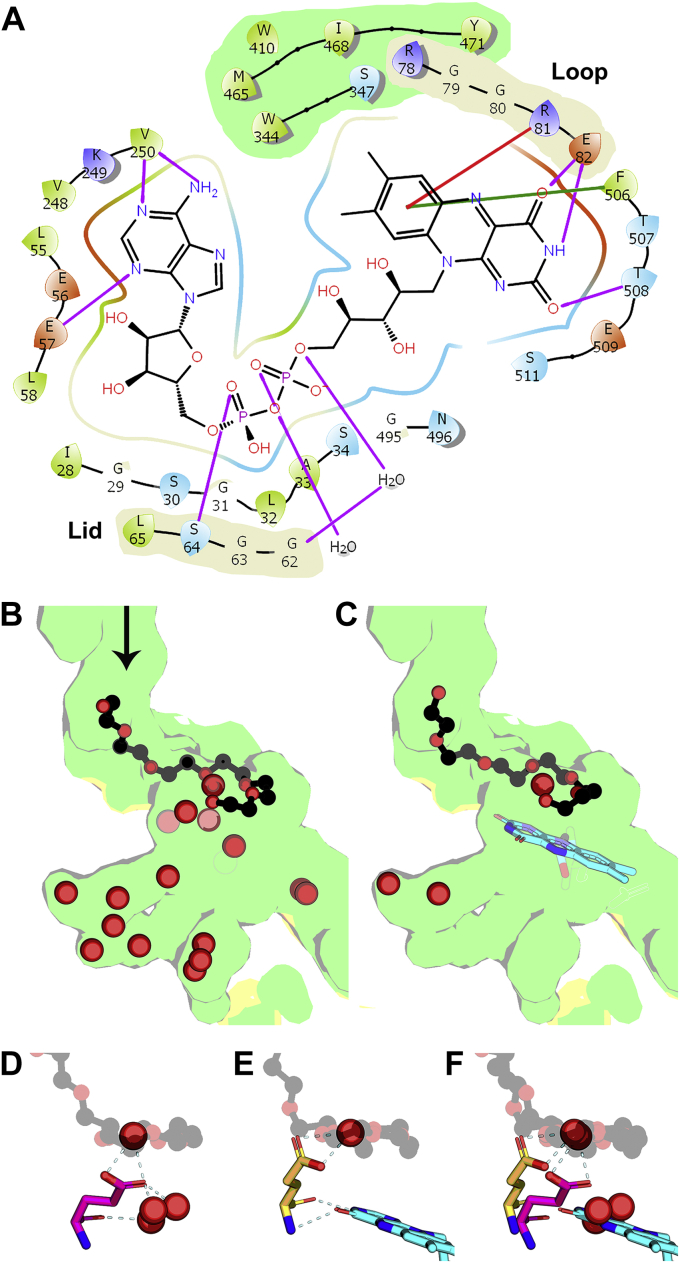
**Organization of the active site by FAD binding.***A*, Schematic of hydrogen bond (*purple lines*), π–π stacking (*green line*), and cation–π (*red line*) interactions between OhyA structural elements and FAD in the OhyA•PEG400•FAD complex with the lid closed (PDB: 7KAW, MolC) created with Maestro. The locations of the catalytic Loop and Lid are highlighted (*beige shading*). The hydrophobic surface accommodating the FAD xylene ring is derived from the fatty acid lobe (*green shading*). *B*, The active site before FAD binding in the OhyA•PEG400 complex (PDB: 7KAV, MolA). The active site is formed by the FAD (*yellow*) and fatty acid (*green*) lobes and contains the substrate analog PEG400 (*black*) and water molecules (*red spheres*). The *arrow* denotes direction of the incoming fatty acid. *C*, The active site with FAD bound in the OhyA•PEG400•FAD complex (PDB: 7KAW, MolB). Same orientation and color coding as Panel B but containing FAD (*cyan*). *D*, Glu82 (*magenta*) interacts with four ordered waters in the OhyA•PEG400 complex (PDB: 7KAV, MolA). *E*, Glu82 (*yellow*) coordinates a single water following FAD (*cyan*) binding in the OhyA•PEG400•FAD complex (PDB: 7KAW, MolB). *Dashed lines* are hydrogen bonds connecting FAD to the backbone carbonyl and amide of Glu82. *F*, Overlay of Panels D and E illustrating the desolvation of the active site, the conformational change in the orientation of Glu82, and the substrate water. FAD, flavin adenine dinucleotide; OhyA, oleate hydratase; PEG400, polyethylene glycol 400.

FAD is a nucleotide cofactor composed of riboflavin (isoalloxazine ring attached to a ribose) linked to adenosine diphosphate (Fig. 4*A*). The isoalloxazine moiety is a tricyclic molecule made up of a hydrophobic xylene ring and a polar uracil ring that are separated by a pyrimidine ring. FAD was soaked into crystals of OhyA•PEG400 to achieve the OhyA•PEG400•FAD ternary complex (PDB: 7KAW). The details of FAD–protein interactions with the catalytic Loop and active site Lid are best visualized in the ternary complex with the active site Lid closed over the FAD (Fig. 4*A*). The xylene ring faces a hydrophobic wall formed by residues contributed by the fatty acid lobe and is stabilized by a π–π stacking interaction with Phe506 and a cation–π interaction (36) with the Arg81 guanidinium group on the catalytic Loop. The uracil nitrogen atom N3 and carbonyl oxygen on carbon atom C4 form hydrogen bond connections to the backbone carbonyl and amide of Glu82, respectively (Fig. 4*A*). The carbonyl oxygen on carbon atom C2 also forms a hydrogen bond with the Thr508 backbone amide. The adenine moiety rests in a leucine and valine–rich hydrophobic cavity. Adenine nitrogen atoms N1 and N10 form hydrogen bond interactions with the Val250 backbone amide and carbonyl, respectively, and adenine nitrogen atom N3 forms a hydrogen bond interaction with the Glu57 backbone amide (Fig. 4*A*). Nucleotide binding proteins often stabilize pyrophosphate binding using a positive helix dipole (37). In OhyA, the FAD pyrophosphate moiety is stabilized by the positive dipole of helix α2 (Fig. S4) and hydrogen bonds with water molecules. The residues that make up helix α2 are located within the dinucleotide-binding motif/phosphate-binding signature sequence found in all glutathione reductase structural family members (35) (Fig. S2). The only direct hydrogen bond contact between the Lid and FAD is the backbone amide of Lid residue Ser64 interacting with a FAD phosphate (Fig. 4*A*). The weak stabilization of the closed Lid conformation explains why electron density for the Lid is most often not observed in OhyA structures.

The OhyA•PEG400 active site (PDB: 7KAV, MolA) in the absence of FAD is exposed to solvent and filled with water molecules (Fig. 4*B*). FAD binding in the OhyA•PEG400•FAD complex (PDB: 7KAW, MolB) squeezes the water molecules out of the active site with the exception of the substrate water (Fig. 4*C*). Glu82 on the catalytic Loop coordinates the substrate water from below the PEG400 substrate analog in the absence of FAD and also forms hydrogen bonds with three additional nearby water molecules in the active site (Fig. 4*D*). When FAD binds, the catalytic Loop conformation is altered by its direct interaction with FAD, and the Glu82 sidechain is repositioned to form hydrogen bonds with the substrate water (Fig. 4*E*). An overlay of these two states shows how FAD displaces the nearby water molecules and rotates Glu82 into a catalytic active conformation connected to a single substrate water molecule (Fig. 4*F*).

### The active site lid

**Figure 5:**
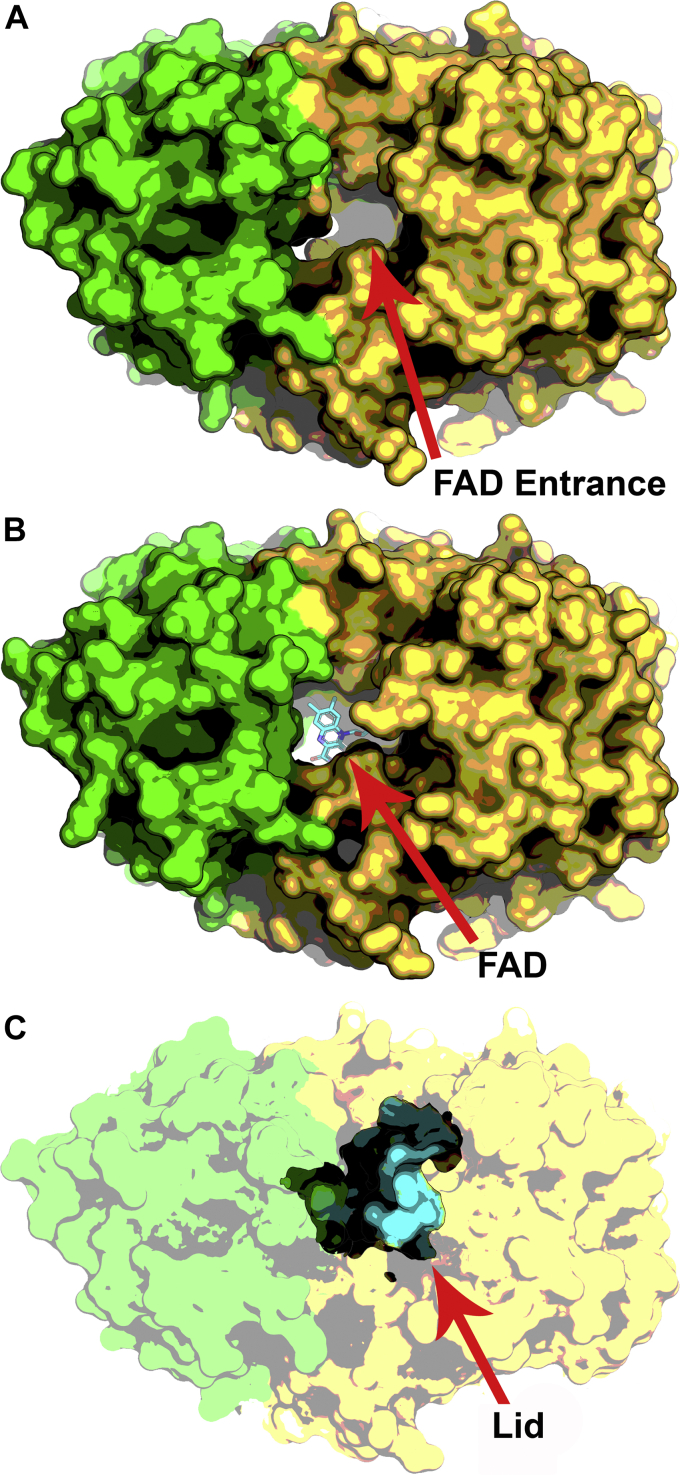
**A flexible lid covers the active site.***A*, Exterior surface rendering of the OhyA•PEG400 complex (PDB: 7KAW, MolA) showing the open lid and the vacant FAD binding cavity exposed to solvent. FAD lobe is yellow, fatty acid lobe is green. OhyA protomers are rotated 90° about the P axis from the view in Figure 2*B*. *B*, FAD (*cyan*) bound at the active site in the OhyA•PEG400•FAD complex with the FAD lid open (PDB: 7KAW, MolB). *C*, The Lid residues (*cyan*) form a 3_10_ helix that closes over the active site in the OhyA•PEG400•FAD complex (PDB: 7KAW, MolC). FAD, flavin adenine dinucleotide; OhyA, oleate hydratase; PEG400, polyethylene glycol 400.

The FAD entrance to the OhyA active site is located at the interface of the FAD and fatty acid lobes along the P axis of the protein (Fig. 5*A*). In this view, the empty active site of the OhyA•PEG400 complex (PDB: 7KAW, MolA) is a gaping solvent-exposed hole in the protein surface, and the Lid that covers the active site is not observed. FAD binds into this cavity, but the Lid remains unstructured in OhyA•PEG400•FAD complex (PDB: 7KAW, MolB) (Fig. 5*B*). In OhyA•PEG400•FAD complex (PDB: 7KAW, MolC), FAD is bound in the pocket, and the Lid has undergone a conformational change from its flexible state to a 3_10_-helix that covers the hole (Fig. 5*C*). Lid closure compresses the active site reducing the distance between Glu82 and PEG400 by >1 Å (Fig. S5). This compression means that the OhyA•PEG400•FAD complex with the Lid closed (PDB: 7KAW, MolC) does not contain the substrate water, but rather Glu82 is now directly hydrogen bonded to the backbone carbonyl of Met186 on the α7 helix dipole (Fig. S5*B*). The *E. meningoseptica* OhyA•PEG400•FAD structure with the Lid closed also does not contain a substrate water molecule in the active site (20). These observations suggest that Lid closure compresses the active site, shortening the distances between the substrate water, Glu82, and the oleate alkene to potentially accelerate catalysis.

### Fatty acid trajectory through the active site

**Figure 6:**
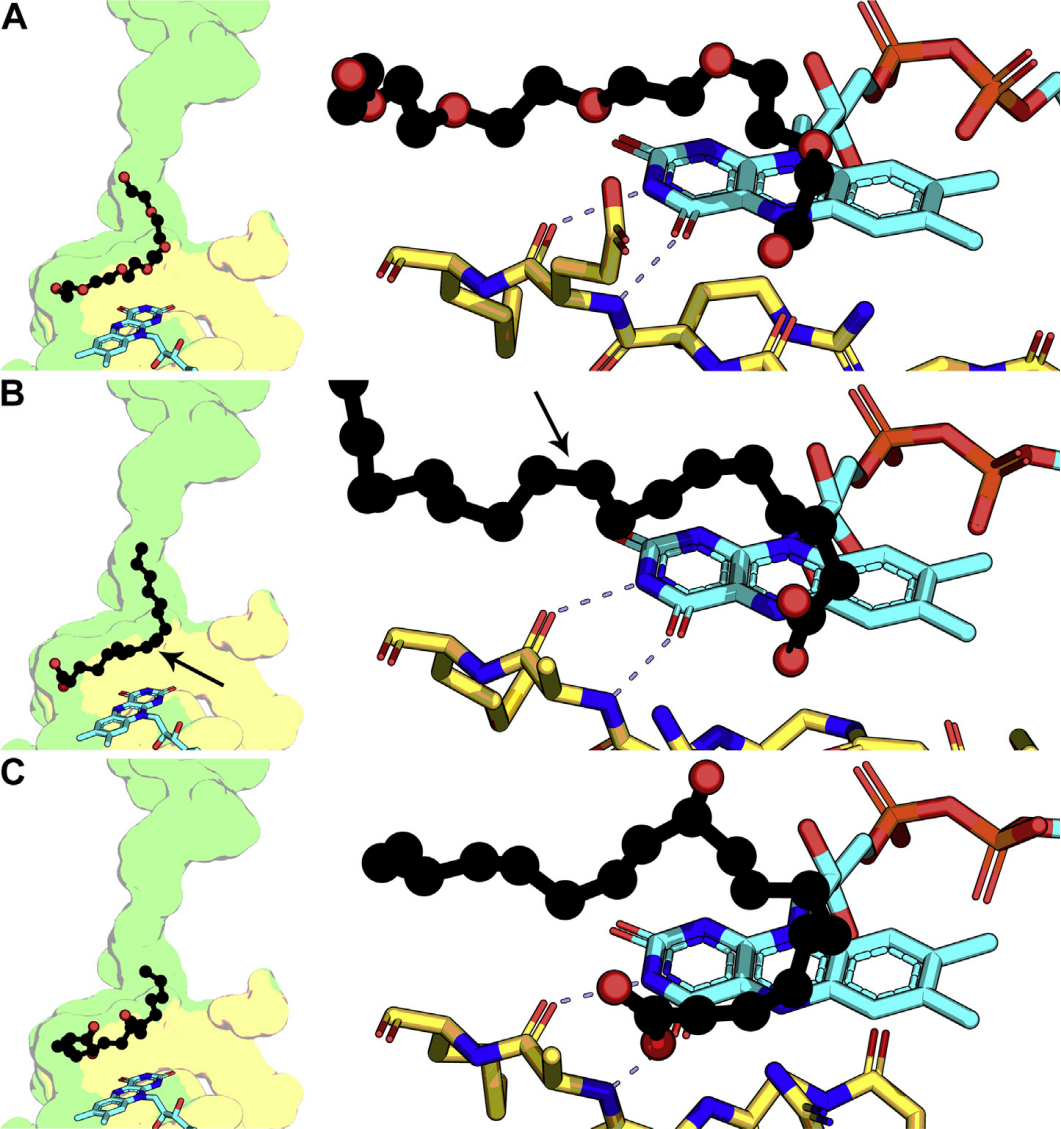
**Substrate, substrate analog, and product complexes.** The substrate tunnel and active site surfaces are derived from the FAD (*yellow*) and fatty acid (*green*) lobes. *A*, Positions of PEG400 (*black*) and FAD (*cyan*) in the active site of the OhyA•PEG400•FAD complex (PDB: 7KAW, MolA). PEG400 curls over FAD which forms two hydrogen bonds with the backbone carbonyl and amide of Glu82 on the catalytic loop (*yellow*). *B*, Oleate (*black*) is released from the tunnel and curls into the active site over FAD (*cyan*) which has the same hydrogen bond interaction with the catalytic loop (*yellow*) in the OhyA(E82A)•oleate•FAD complex (PDB: 7KAZ, MolB). *Arrow* denotes the position of the Δ9 double bond. *C*, Positions of the 10-hydroxystearate (*h*18:0) product (*black*) in relation to FAD (*cyan*) and the catalytic loop (*yellow*) in the OhyA(E82A)•*h*18:0•FAD product complex (PDB: 7KAZ, MolC). FAD, flavin adenine dinucleotide; OhyA, oleate hydratase; PEG400, polyethylene glycol 400; h18:0, (R)-10-hydroxyoctadecanoic acid.

Comparison of the positions of PEG400, oleate, and *h*18:0 product shows that PEG400 is a nonreactive substrate analog that adopts the same curled conformation in the active site as the substrate and product (Fig. 6). The PEG400 substrate analog and oleate substrate positions are nearly identical while FAD remains in the same position and maintains the same backbone hydrogen bond interactions with the catalytic Loop across all substrate and product complexes (Fig. 6, *A* and *B*). Oleoyl alcohol is an OhyA substrate (23) and the position of the terminal hydroxyl of PEG400 is a model for the location of the oleoyl alcohol hydroxyl or oleate carboxyl just before catalysis occurs. The OhyA•product•FAD complex (Fig. 6*C*) shows that the hydroxy-fatty acid product is also located in the same place except it has rotated further around the active site. These data indicate that the terminal carboxyl of oleate enters the active site to participate in hydronium ion formation through formation of hydrogen bonds with Glu82 as observed in the OhyA•PEG400•FAD complex (see below).

**Figure 7:**
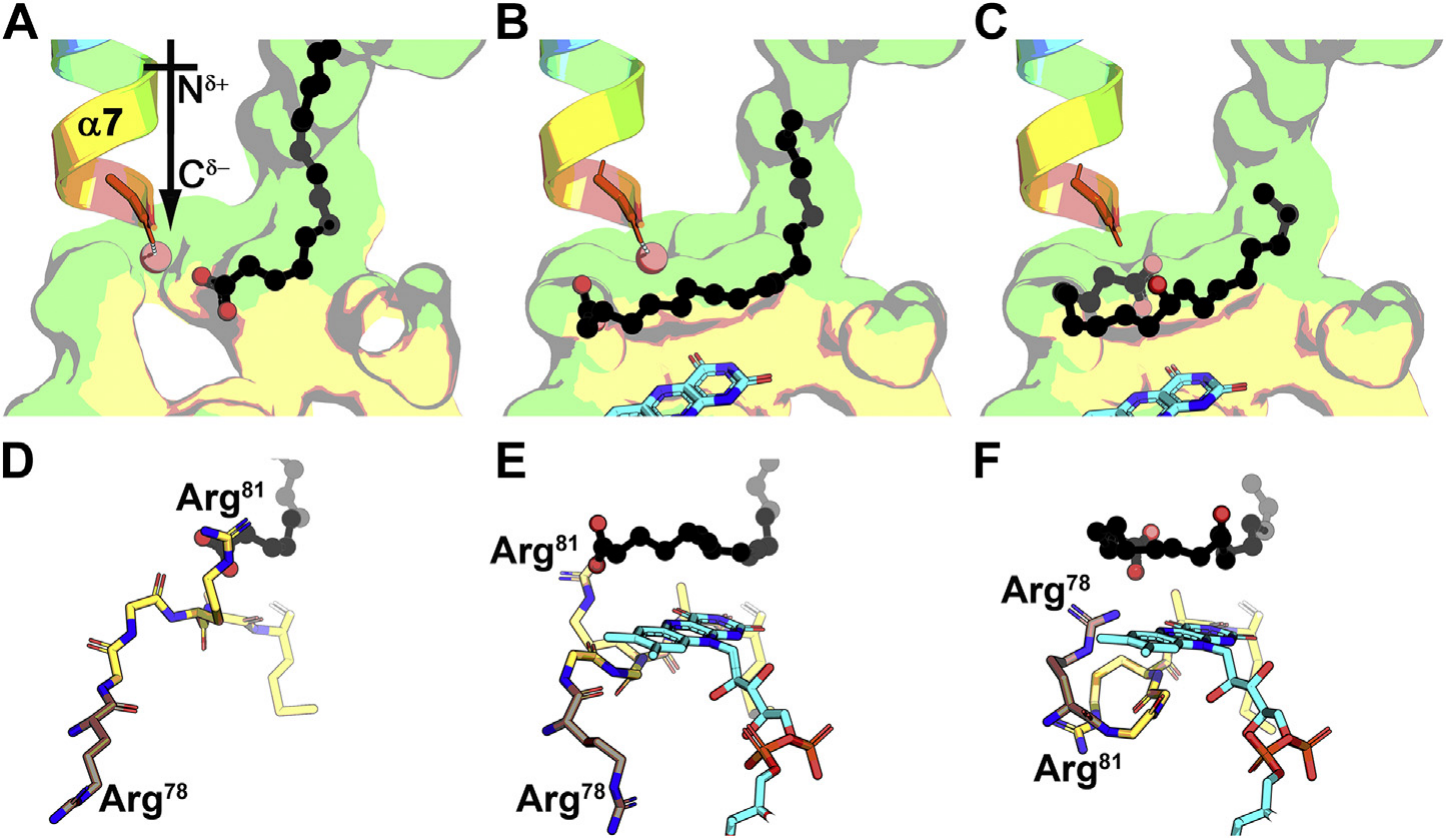
**Progression of oleate into the active site.** Oleate is *black*, active site loop residues are *yellow*, and FAD is *cyan*. The active site surface is derived from the FAD (*yellow*) and fatty acid (*green*) lobes. The α7 helix dipole is colored from *blue* (amino terminus) to *red* (carboxy terminus). *A*, The OhyA(E82A)•oleate complex (PDB: 7KAZ, MolA) shows the α7 helix dipole interacting with the water substrate (*red sphere*). *B*, The OhyA(E82A)•oleate•FAD complex (PDB: 7KAZ, MolB) shows FAD interactions with the catalytic loop rotates Arg81 out of the tunnel, and the oleate is released to curl into the active site. The substrate water molecule is anchored by the α7 helix dipole and oleate makes van der Walls contact with the FAD ring system. *C*, The OhyA(E82A)•*h*18:0•FAD product complex (PDB: 7KAZ, MolC) shows the substrate water transferred to the 10-carbon of the substrate and the acyl chain curled further into the active site above the FAD ring system. *D*, Oleate is prevented from entering the active site by Arg81 that blocks the tunnel and forms direct hydrogen bond contact with the oleate carboxyl group. *E*, Arg81 rotates out of the tunnel to allow the oleate to move into the active site. *F*, The product complex shows the catalytic loop is in a different conformation with Arg78 (*brown*) adjacent to the fatty acid carboxyl and Arg81 is rotated to point away from the fatty acid. FAD, flavin adenine dinucleotide; OhyA, oleate hydratase; PEG400, polyethylene glycol 400; h18:0, (R)-10-hydroxyoctadecanoic acid.

The wall created by Arg81 between the tunnel and the active site prevents oleate from entering the active site in the OhyA(E82A)•oleate complex (Fig. 7*A*). The catalytic water molecule is present and coordinated by a helix dipole from α7 that ends in the backbone carbonyl of Met186 (Fig. 7*A*). The direction of the helix dipole focuses a partial negative charge on the substrate water, which stably coordinates the water even though Glu82 is missing from the active site (Fig. S4). FAD binding engages the backbone of the catalytic Loop and flips Arg81 out of the tunnel allowing oleate to rotate into the active site (Fig. 7*B*). The FAD-induced conformational change creates a continuous surface creating a donut-shaped active site. The *h*18:0 product has advanced ∼4 to 5 Å into the active site and curls around the doughnut shaped cavity (Fig. 7*C*). It remains unclear how the *h*18:0 exits the protein. The orientation of the product suggests it may exit by moving up the fatty acid tunnel. However, when FAD leaves the active site, there is a large opening that the more polar hydroxylated product may diffuse out.

### Movements of the catalytic loop

The Loop contains two arginine residues (Arg78 and Arg81) whose movements direct the fatty acid around the active site doughnut. Before FAD binding, Arg81 walls off the fatty acid in the substrate tunnel from access to the active site as the Arg81 side chain hydrogen bonds with the oleate carboxylate (Fig. 7*D*). Arg78 is pointed away from the oleate. FAD binding engages the catalytic Loop backbone (Fig. 6) triggering the Arg81 side chain to shift 8 Å from its upright conformation releasing the fatty acid into the active site (Fig. 7*E*). In this new conformation, the Arg81 side chain is still hydrogen bonded to the fatty acid carboxylate pulling the acyl chain into the active site torus (Fig. 7*E*). The Arg78 side chain is still oriented away from the active site and does not interact with oleate substrate (Fig. 7*D*). However, in the *h*18:0 product complex, the catalytic Loop arginine residues flip positions (Fig. 7*F*). Arg78 side chain swivels 11 Å toward *h*18:0 and the Arg81 side chain turns an additional 8 Å and now points away from the active site. These conformational changes place Arg78 behind the fatty acid carboxyl to facilitate the continued rotation of the product around the active site. These data reveal the subtle changes in the catalytic Loop residues that guide the oleate through the active site as the reaction proceeds.

### Role of Tyr201

**Figure 8:**
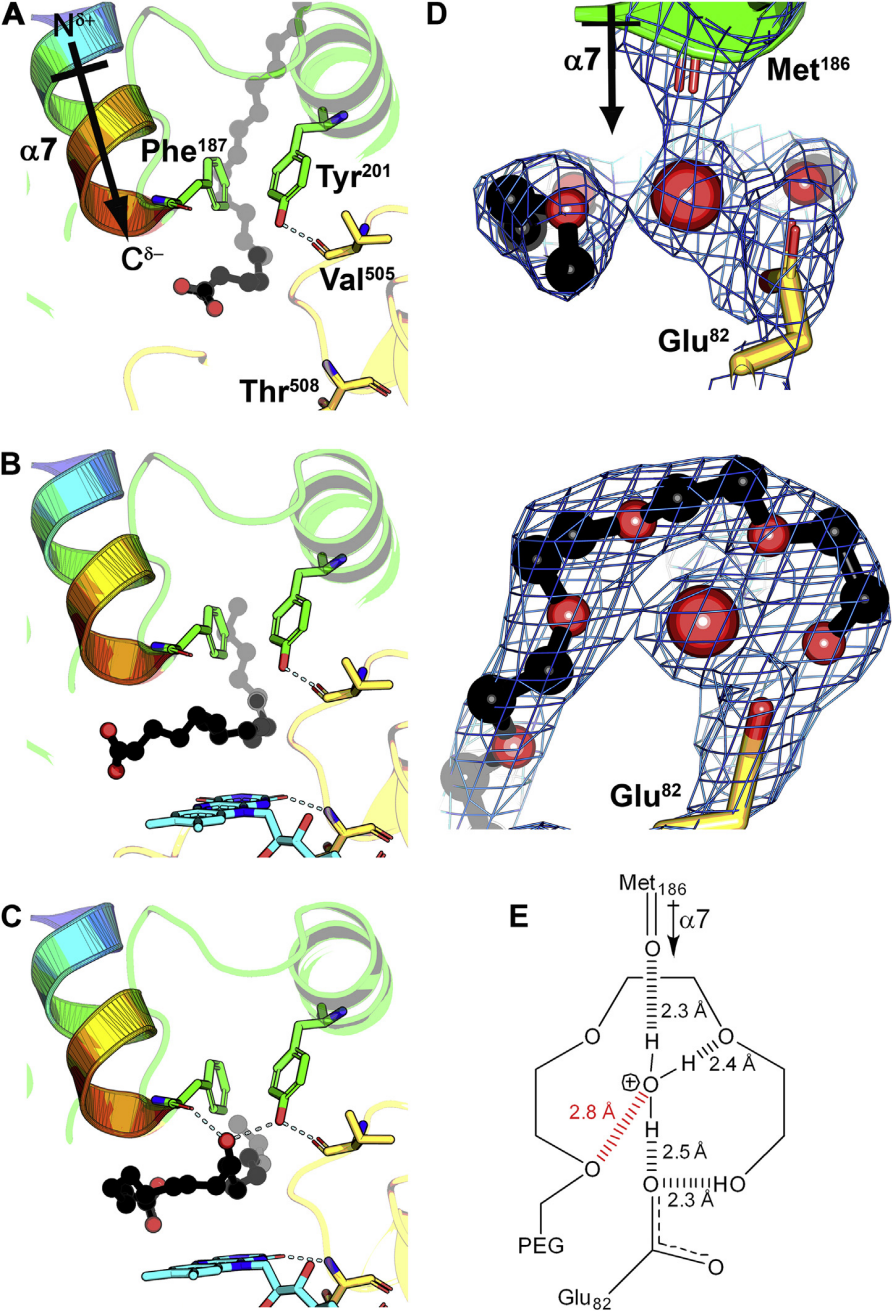
**Role of Tyr201 and the hydronium ion.** Contributions from the FAD lobe are yellow, fatty acid lobe is green, fatty acid is black, and FAD is cyan. *A*, The OhyA(E82A)•oleate complex (PDB: 7KAZ, MolA) shows the α7 helix dipole colored from blue (amino terminus) to red (carboxy terminus). Tyr201 on the fatty acid lobe makes a 2.7 Å hydrogen bond interaction with the carbonyl of Val505 on the FAD lobe to seal one side of the active site. *B*, The OhyA(E82A)•oleate•FAD complex (PDB: 7KAZ, MolB) shows the oleate chain rotating into the active site above FAD making van der Waals contact with the hydrophobic xylene ring. *C*, The OhyA(E82A)•*h*18:0•FAD product complex (PDB: 7KAZ, MolC) shows the Phe187 backbone carbonyl-product hydroxyl-Tyr201 hydroxyl-Val505 backbone carbonyl hydrogen bond network that stabilizes water addition to carbon-10 of the fatty acid. *D*, Two rotated snapshots of the coordination network show three direct interactions with contiguous electron density to the catalytic water molecule (*red sphere*). An interaction between Glu82 and the PEG400 terminal hydroxyl is evident. Electron density calculated from a 2mF_O_ − DF_C_ map contoured at 1 σ (*blue mesh*). *E*, Diagram of the hydrogen bonding network surrounding the substrate water. Distances represent measurements between oxygen atoms are shown with three hydrogen bonds connected to the water oxygen indicating it is a positively charged hydronium ion. FAD, flavin adenine dinucleotide; OhyA, oleate hydratase; PEG400, polyethylene glycol 400; h18:0, (R)-10-hydroxyoctadecanoic acid.

Mutagenesis experiments suggested a conserved tyrosine (Tyr201 in OhyA) has a role in catalysis and was proposed to house the proton abstracted by the substrate *cis* double bond (20). Tyr201 is in the fatty acid lobe and the tyrosyl oxygen forms a 2.7 Å hydrogen bond interaction with the backbone carbonyl of Val505 on the FAD lobe to seal one side of the active site (Fig. 8*A*). The almost contiguous electron density across Tyr201 and Val505 in all structures reflects the strength of this interlobe intramolecular hydrogen bond. This Val505 interaction fixes the position of a structured loop that makes a π–π stacking interaction between the Phe506 and the FAD xylene ring and a hydrogen bond between the Thr508 backbone amine and the FAD uracil ring (Fig. 4*A*). These structural roles for Tyr201 account for the lower thermal stability of OhyA(Y201F) (Fig. 1*B*). The Tyr201–Val505 hydrogen bond connection is intact after oleate enters the active site (Fig. 8*B*) and is observed in all OhyA structural complexes (Fig. 8) making the Tyr201 hydroxyl proton unavailable to participate in catalysis. This means Tyr201 does not donate a proton to carbon 9 of oleate to initiate the OhyA reaction. Rather than participate in catalysis, the Tyr201 hydroxyl forms a hydrogen bond network that stabilizes the hydroxy-fatty acid product (Fig. 8*C*). The *h*18:0 hydroxy participates in a Phe187–*h*18:0–Tyr201–Val505 hydrogen bond network (Fig. 8*C*). Water addition to the carbocation intermediate generates a product that would exactly fit into this network. OhyA production is stereoselective, and we determined if this selectivity was altered in the OhyA(Y201F) mutant. Mass spectrometry confirmed carbon-10 is the position for hydroxylation for OhyA and OhyA(Y201F) (Fig. S6). Product stereochemistry was determined by derivatization and chiral chromatography (Fig. S7), and (*R*)-10-hydroxyoctadecanoic acid was the sole product in both OhyA and OhyA(Y210 F). Loss of the Tyr201 tyrosyl oxygen in OhyA(Y201F) disrupts the active site hydrogen bond network that stabilizes the OhyA active site structure and the hydroxy-fatty acid product.

### Hydronium ion

The acid-catalyzed electrophilic hydration of alkenes proceeds by the removal of a proton from a hydronium ion followed by addition of water to the transient carbocation intermediate (29). Rotated views show the electron density contoured at 1 σ in the active site of the OhyA•PEG400•FAD substrate analog complex (PDB: 7KAW, MolB) (Fig. 8*D*). The substrate water is the focus of a hydrogen bond network that includes the Glu82 side chain, the backbone carbonyl of Met186 from the α7 helix dipole, and the hydroxyl of PEG400 (Fig. 8*D*). The contiguous electron density around the substrate water reflects the strength of the hydrogen bonds and leads to the conclusion that a buried hydronium ion is created at the active site (Fig. 8*E*). Curling the substrate around the hydronium ion positions the 9-carbon of the hydrocarbon chain within 2.8 Å of the substrate water (Fig. 8*E*). This network is configured to push the acidic proton of Glu82 onto the water to create a hydronium ion substrate that is attacked by the fatty acid alkene, followed by the addition of water to the carbocation formed at carbon-10.

## Discussion

**Figure 9:**
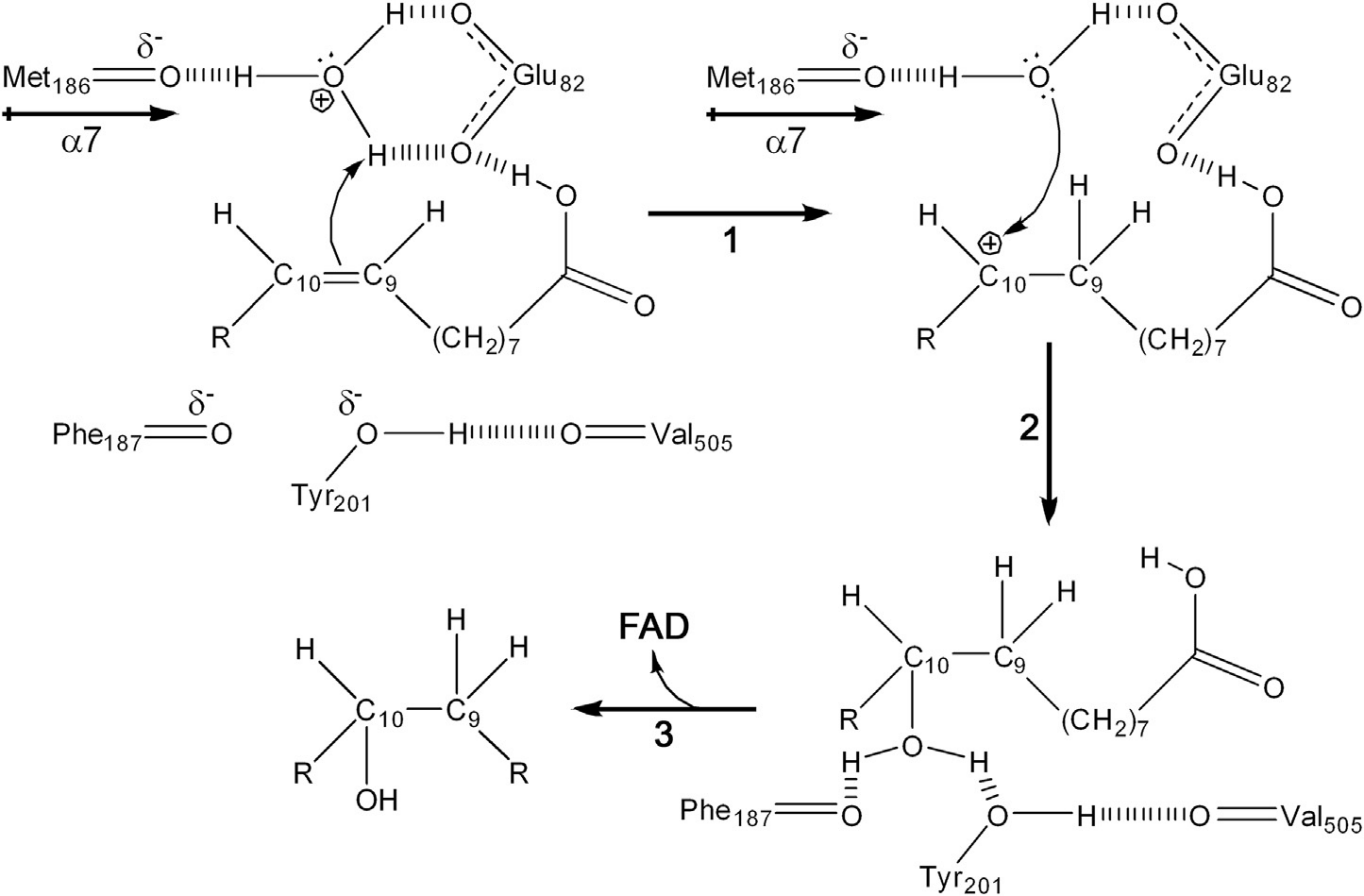
**Acid-catalyzed alkene hydration by OhyA.** FAD binding leaves a buried water in the active site where the α7 dipole, Glu82 and the oleate carboxyl create an environment that stabilizes the hydronium ion (H_3_O^+^). The acidic water proton (H^+^) is attacked by the oleate π bond to form a planar carbocation as a transient intermediate (**1**). Water then attacks the carbocation to form an oxonium water adduct that is stabilized by the hydrogen bond network involving Tyr201, Phe187 and Val505 (**2**). The active site lid opens, FAD leaves and the proton is released to the hydrated active site leading to product formation (**3**). FAD, flavin adenine dinucleotide; OhyA, oleate hydratase; PEG400, polyethylene glycol 400.

Our analyses illustrate how multiple structural elements of OhyA combine to accelerate catalysis using the acid-catalyzed chemical mechanism for nonactivated alkene hydration (29, 38) (Fig. 9). The first step is the electrophilic addition of a hydrogen ion (H^+^) to the isolated, unpolarized double bond. This is different than more common biochemical reactions where water acts as a nucleophile to attack α,β-unsaturated (Michael) acceptors, like conjugated or polarized double bonds. The first requirement for the OhyA reaction is the creation of a hydrogen ion electrophile (H_3_O^+^) that can be attacked by the alkene to form a carbocation intermediate. In the OhyA•oleate state, oleate is excluded from the active site that is filled with water by Arg81. The critical functions of FAD binding are to (1) exclude of all but one water from the active site and (2) engage the active site Loop to position Glu82 with respect to the water and rotate Arg81 away to allow the oleate to enter the active site torus. The OhyA•PEG400•FAD substrate analog complex shows a hydrogen bond network in the electron density surrounding the buried active site water that is consistent with a hydronium ion (Fig. 9). The buried hydronium ion is stabilized by an interaction with the negative α7 helix dipole mediated by the carbonyl of Met186, both oxygens of the Glu82 carboxyl, and the terminal hydroxyl of PEG400. The importance of Glu82 in creating the hydronium ion is evidenced from the severely compromised catalytic activity of OhyA(E82A). It is instructive that OhyA(E82A) activity is not zero illustrating that positioning of the buried water by the α7 dipole and the arrival of the protonated substrate carboxyl at the active site also contribute to catalysis. Curling oleate around the substrate water positions the carboxyl to participate in catalysis by bringing another acid to the active site to stabilize the hydronium ion (Fig. 9). Fatty acid esters and alkenes are not OhyA substrates (39), but oleoyl alcohol is (23), consistent with a role for the substrate as a hydrogen bond donor in the reaction. Overlaying oleoyl alcohol on the structure of the PEG400 substrate analog complex positions the C9 atom within 2.8 Å of the hydronium ion to facilitate catalysis (Fig. 8*E*). 1-Decene is hydroxylated by OhyA in the presence of a short-chain fatty acid acting as a spacer (40) providing additional direct support for the protonated substrate carboxyl in promoting the reaction. Carboxylic acids are typically protonated when they are buried in the protein interior with the average pKa rising from 4.5 to 7.7 (41). Modeling Glu82 and oleate as protonated species in our OhyA structures is consistent with their observed isolation from bulk water. Thus, multiple protein and substrate interactions collaborate to accelerate catalysis by forming a buried hydronium ion at the active site.

With the hydronium ion properly positioned, H^+^ adds to the π bond of oleate creating a tetrahedral sp^3^ hybridized C9 atom and a planar sp^2^ carbocation intermediate on the C10 atom and a water (Fig. 9). The water next attacks the empty *p*-orbital of the carbocation from either side leading to a racemic mixture of products. In OhyA, the position of the single water in the active site is fixed, meaning the reaction pathway leads to a stereospecific outcome. Tyr201 is a second residue that impacts catalysis by stabilizing the formation of the oxonium water adduct to the C10 atom (Fig. 9). The Tyr201 hydrogen bond network visualized in the OhyA•*h*18:0•FAD product complex engages both protons of the attacking water using a network consisting of the Phe187 carbonyl (influenced by the α7 helix dipole), the product hydroxyl oxygen, the tyrosyl oxygen of Tyr201, and the carbonyl of Val505 (Fig. 8*C*). In the final step, the oxonium intermediate loses a hydrogen ion to solvent resulting in an alcohol. Our data match these established steps in the chemical hydration of an alkene but are not consistent with the prevailing proposal that the proton on the Tyr201 hydroxyl attacks the double bond in the first step (20, 23, 30, 31, 32). The modest reduction in enzyme activity in OhyA(Y201F) is not consistent with a role for Tyr201 in initiating the reaction, and there is no chemical precedent for this reaction pathway. The hydroxyl proton of Tyr201 is clearly pinned to the backbone carbonyl of Val505 and is not available (Fig. 8*C*). This important hydrogen bond connects the fatty acid and FAD lobes, positions the polar loop to interact with FAD (Phe506 & Thr508), and seals one side of the active site (Fig. 3*A*). The lower thermal stability of OhyA(Y201F) confirms a structural role for the Tyr201–Val505 interaction. Our OhyA substrate and product complexes show the location of the oleate carboxyl and double bond in the OhyA active site. These locations are significantly different (∼7–9 Å) than those predicted by the docking model currently used to design site-directed mutants in protein engineering experiments (23, 32, 40). There is considerable interest in the use of OhyA as a biocatalyst to create stereospecific hydroxylated fatty acids (20, 23, 30, 31, 32, 39, 40), and our new structural information provides a solid framework for engineering more versatile biocatalysts.

## Experimental procedures

### Materials

All chemicals and reagents were obtained from Sigma-Aldrich or Fisher Scientific unless otherwise indicated.

### Cloning, expression, and purification of OhyAs

The *S. aureus* *ohyA* DNA sequence was synthesized and cloned into pET28a with an N-terminal His tag for purification from *Escherichia coli* as previously described (15) to form pOhyA. The OhyA(E82A) mutation was introduced by modifying pOhyA using the QuikChange Lightning site-directed mutagenesis kit (Agilent Technologies, Cat# 210519) and primers OhyA(E82A)-F (5'-CCGCGGTGGTCGTGCAATGGAGAACCACT-3') and OhyA(E82A)-R (5'-AGTGGTTCTCCATTGCACGACCACCGCGG-3') to form pOhyA(E82A). The OhyA(Y201F) mutation was introduced by modifying pOhyA using the QuikChange Lightning site-directed mutagenesis kit (Agilent Technologies, Cat# 210519) and primers OhyA(Y201F)-F (5'-TCTGCAATGGAAATGCGTCGCTTTCTAATGCGATTC-3') and OhyA(Y201F)-R (5'-GAATCGCATTAGAAAGCGACGCATTTCCATTGCAGA-3') to form pOhyA(Y201F). The pOhyA, pOhyA(E82A), and pOhyA(Y201F) plasmids were transformed into *E. coli* BL21(DE3) cells (Millipore Sigma, Cat# 70,235), and isolates were obtained on Luria broth agar imbedded with 50 μg/μl kanamycin (Gold Biotechnology, Cat# K-120). Transformants were amplified in Luria broth containing 50 μg/μl kanamycin (Gold Biotechnology, Cat#K-120) and shaken at 37 °C, 200 rpm. Cells were grown to an OD_600_ of 0.6 and then cooled to 16 °C before induction with 1 mM isopropyl-β-D-thiogalactoside (Gold Biotechnology, Cat# I2481 C) overnight. Cells were harvested and then lysed in buffer containing 20 mM Tris, pH 8.0, 10 mM imidazole, 200 mM NaCl, and a dissolved tablet of Pierce protease inhibitor (Thermo Fisher Scientific, Cat# A32955). The OhyA, OhyA(E82A), and OhyA(Y201F) proteins were separated from cell lysates by nickel agarose beads (Gold Biotechnology, Cat# H-320) and eluted in buffer containing 20 mM Tris, pH 8.0, 250 mM imidazole, and 200 mM NaCl. The eluant was gel filtered into buffer containing 20 mM Tris, pH 8.0, 200 mM NaCl using the HiLoad Superdex 200 column (Cytiva Life Sciences, Cat# 28989335) with dimensions 16 mm x 60 cm. The molecular weights of the OhyA, OhyA(E82A), and OhyA(Y201F) proteins were estimated using an XBridge BEH SEC 200 Å 3.5 μm column (Waters Corporation, Cat# 176003595) with dimensions 7.8 mm x 150 mm. The standard curve for the XBridge BEH SEC column used thyroglobulin (669 kDa), immunoglobin (150 kDa), BSA (66.4 kDa), and myoglobulin (17 kDa).

### OhyA activity assay

The OhyA, OhyA(E82A), and OhyA(Y201F) activities were assayed as described previously (15). Briefly, 20 μl reactions containing 50 mM potassium phosphate buffer, pH 6.0, 150 mM NaCl, 10 mM DTT, 50 μM FAD (Millipore Sigma, Cat# F6625), 0.2 mg/ml fatty acid-free bovine serum albumin (Millipore Sigma, Cat# A6003), 20 μM [1-^14^C]oleate (specific activity 54.3 mCi/mmol) (PerkinElmer, Cat# NEC317250UC), and 1 μg OhyA, 100 μg OhyA(E82A), or 10 μg OhyA(Y201F) were incubated at 37 °C for 20 min. These enzyme concentrations were experimentally determined based on prior reactions with varying enzyme concentration monitoring detectable hydroxy fatty acid product formation. A 10 μl aliquot of the reactions were spotted on a Silica Gel H thin-layer plate (Spectrum Chemical, Cat# 476–10,450) and separated using chloroform:methanol (90:10, v/v). Hydroxy fatty acid product formation was visualized using a Typhoon PhosphorImager and quantified using ImageQuant (Cytiva Life Sciences, https://www.cytivalifesciences.com/en/us/shop/protein-analysis/molecular-imaging-for-proteins/imaging-software).

### OhyA product stereochemistry

OhyA and OhyA(Y201F) activity assays were performed with oleate (Matreya, LLC, Cat# 1022), the fatty acids extracted and the solvent removed under nitrogen (42). The position of hydroxylation was determined by product scans operating a Sciex QTrap 4500 mass spectrometer in the negative mode and compared with (*R*)-9-hydroxyoctadecanoic acid (Larodan Research Grade Lipids, Cat# 14–1809) and (*R/S*)-10-hydroxyoctadecanoic acid (AA Blocks, LLC, Cat# AA0032L4) standards as described previously (15). The source parameters were ion spray voltage, −4500 V; curtain gas, 15 p.s.i.; temperature, 250 °C; collision gas, high; ion source gas 1, 15 p.s.i.; ion source gas 2, 20 p.s.i.; declustering potential, −25 V; and collision energy, −35 V.

The enantiomer composition was determined by derivatization and chiral chromatography. The (*R/S*)-10-hydroxyoctadecanoic acid (15 mg, 0.047 mmol, 1 eq) (AA Blocks, LLC, Cat# AA0032L4) was combined with HBTU (O-(Benzotriazol-1-yl)-*N*,*N*,*N*′,*N*′-tetramethyluronium hexafluorophosphate) coupling reagent (21 mg, 0.056 mmol, 1.2 eq) (Oakwood Chemical, Cat# 009019) and placed under a nitrogen atmosphere. The reaction was diluted with 0.250 ml of tetrahydrofuran (Millipore Sigma, Cat# 401757) then treated with (*R*)-α-methylbenzylamine (6.5 μl, 0.051 mmol, 1.1 eq) (Millipore Sigma, Cat# 115541) followed by diisopropylethylamine (12 μl, 0.070 mmol, 1.5 eq) (Millipore Sigma, Cat# D125806) at room temperature. The reaction was stirred overnight then diluted with 0.5 ml of methanol and eluted through a short plug of SiliaBond-Carbonate (0.5 g) (Silicycle, Cat# R60430B) stacked onto a short plug of SiliaBond-Tosic Acid (0.5 g) (Silicycle, Cat# R66030B) with the aid of an additional 3 ml of methanol by gravity. The collected filtrate was concentrated, and the resulting white solid was dissolved in methanol at 2 mg/ml suitable for chiral chromatography. Fatty acids from the enzyme reaction were derivatized using the same procedure and injected directly onto the column after solvent switch from tetrahydrofuran (Millipore Sigma, Cat# 401757) to methanol. Chiral chromatography was performed on a Waters UPC2 supercritical fluid chromatography system on a Chiral Technologies Chiralpak IG (4.6 mm x 150 mm) column (Chiral Technologies, Cat# 87,324) using an isocratic 30% methanol/supercritical carbon dioxide mobile phase. Analyte peaks appeared at 6.2 (*R*) and 6.6 min (*S*).

### Fatty acid association

The OhyA protein was assayed to determine fatty acid binding ability using tritium labeled oleic acid. Briefly, 100 μl reactions containing 50 mM potassium phosphate buffer, pH 6.0, 150 mM NaCl, 1% v/v dimethyl sulfoxide, 20 μM OhyA, and 1 μM [9,10-^3^H]oleate (specific activity 54.0 Ci/mmol) (PerkinElmer, Cat# NET289001MC) were mixed and incubated at 37 °C for 1 h. A 50 μl aliquot of the reaction was separated using an XBridge BEH SEC 200 Å 3.5 μm column (Waters Corporation, Cat# 176003595) with dimensions 7.8 mm x 150 mm. The elution was collected in 20 s increments, and the fractions were analyzed by scintillation counting.

### Thermal stability

The nanoDSF Prometheus NT.48 was used to directly measure dynamic light scattering of OhyA, OhyA(E82A), and OhyA(Y201F). Briefly, 20 μl samples containing 50 mM potassium phosphate buffer, pH 6.0, 150 mM NaCl and 10 μM OhyA, OhyA(E82A), or OhyA(Y201F) were mixed and loaded into Prometheus NT.48 Series nanoDSF Grade High Sensitivity Capillaries (NanoTemper Technologies, Cat# PR-C006) by capillary force action. The capillaries were heated from 20 °C to 90 °C at a rate of 1 °C/min, and the UV light scattering was recorded to determine the apparent aggregation and T_agg_.

### Crystallization and structure determination

The OhyA protein was concentrated to 20 mg/ml for crystallization. Initial screening was performed at 20 °C against the Protein Complex Suite (NeXtal Biotechnologies, Cat# 130715) by hanging drop vapor diffusion method combining 200 nl protein and 200 nl precipitant. Diffraction quality crystals were obtained by combining 1.5 μl protein and 1.5 μl 20% v/v PEG400, 100 mM calcium acetate, and 100 mM MES, pH 6.0. Crystals were cryo-protected with 20% v/v PEG400, 100 mM calcium acetate, 100 mM MES, pH 6.0, and 30% glycerol and then flash-frozen in liquid nitrogen for X-ray diffraction experiments. OhyA was solved by the molecular replacement method using the program Phaser (43) and the coordinates of *E. meningoseptica* OhyA (PDB: 4UIR) (20) as the search model. These crystals contained the OhyA•PEG400 binary complex. The OhyA•PEG400•FAD ternary complex was achieved by soaking OhyA•PEG400 crystals in 20 μl 20% v/v PEG400, 100 mM calcium acetate, 100 mM MES, pH 6.0, and 750 μM FAD (Millipore Sigma, Cat# F6625) overnight at room temperature. Crystals were cryo-protected in 20% v/v PEG400, 100 mM calcium acetate, 100 mM MES, pH 6.0, and 30% glycerol and then flash-frozen in liquid nitrogen. Attempts to achieve the OhyA•FAD binary or OhyA•PEG400•FAD ternary complex by co-crystallization failed because of FAD precipitation. Attempts to deliver fatty acid in the crystallization drop using common agents that solubilize hydrophobic compounds including detergents and dimethyl sulfoxide failed. The OhyA(E82A)•oleate binary complex was achieved by incubating 7 mg/ml (100 μM) OhyA(E82A) with 1 mg/ml fatty acid-free bovine serum albumin (Millipore Sigma, Cat# A6003) and 500 μM oleate (Matreya, LLC, Cat# 1022) for 3 days in 150 mM sodium chloride and 50 mM potassium phosphate, pH 6.0 at room temperature prior to crystallization. The mixture was screened at 20 °C against the PACT Suite (NeXtal Biotechnologies, Cat# 130718) by hanging drop vapor diffusion method combining 200 nl protein and 200 nl precipitant. Diffraction quality crystals were obtained by combining 1.5 μl protein and 1.5 μl 15% v/v PEG3000, 200 mM magnesium chloride, and 100 mM sodium cacodylate, pH 6.5. Crystals were cryoprotected with 15% v/v PEG3000, 200 mM magnesium chloride, 100 mM sodium cacodylate, pH 6.5, and 30% glycerol and then flash-frozen in liquid nitrogen. The OhyA(E82A) substrate-free structure was solved using the same method but omitting oleate. The OhyA(E82A)•oleate•FAD ternary complex was achieved soaking crystals containing OhyA(E82A)•oleate in 20 μl 15% v/v PEG3000, 200 mM magnesium chloride, 100 mM sodium cacodylate, pH 6.5, and 750 μM FAD (Millipore Sigma, Cat# F6625) overnight at room temperature. Crystals were cryoprotected with 15% v/v PEG3000, 200 mM magnesium chloride, 100 mM sodium cacodylate, pH 6.5, and 30% glycerol, flash-frozen again in liquid nitrogen, and X-ray diffraction data were re-collected. Crystals containing OhyA•PEG400 and OhyA(E82A)•oleate that were soaked with FAD turned bright yellow, which is consistent with incorporation of the oxidized cofactor. All diffraction datasets were collected at the SER-CAT beamline 22-ID at the Advanced Photon Source and processed using KYLIN (44) and HKL2000 (45). The structures of the OhyA•PEG400•FAD ternary complex, OhyA(E82A)•oleate binary complex, and OhyA(E82A)•oleate•FAD ternary complex were solved by molecular replacement using the OhyA•PEG400 binary complex structure as a search model. The structures were completed by iterative rounds of refinement using phenix.refine (46) and manual rebuilding using Coot (47). The refinement was monitored by following the R_free_ value calculated from a random subset (5%) of omitted reflections. A summary of the data processing and structure refinement statistics is provided in Table 1. The figures related to protein structures were generated with PyMOL (48).

### In-silico analyses

Surface areas were calculated in PyMOL (48) as previously described (49). The buried surface area by dimerization was calculated by subtracting the surface area of two protomers linked along the Q axis from the combined surface area of the two individual protomers.

### Quantification and statistical analysis

Quantification of activity data was performed using ImageQuant (Cytiva Life Sciences, https://www.cytivalifesciences.com/en/us/shop/protein-analysis/molecular-imaging-for-proteins/imaging-software), and data analysis (i.e., calculation of mean and standard error) was performed using PRISM (GraphPad Software, https://www.graphpad.com/scientific-software/prism/).

## Data availability

The structure factors and atomic coordinates of OhyA•PEG400, OhyA•PEG400•FAD, OhyA(E82A), OhyA(E82A)•oleate, OhyA(E82A)•oleate•FAD have been deposited to the Protein Data Bank (PDB) under the accession codes of 7KAV, 7KAW, 7KAX, 7KAY, and 7KAZ, respectively.

## Conflict of interest

The authors declare that they have no conflicts of interest with the contents of this article.
