# Supplementary material for: Structure and mechanism of Staphylococcus aureus oleate hydratase (OhyA)
Source: J Biol Chem. 2021 Jan 9;296:100252. doi: 10.1074/jbc.RA120.016818 (PMC7948970; doi:10.1074/jbc.RA120.016818)
Supplement: Figures S1–S7 [file mmc1.pdf]

Supplemental Figures

Structure and mechanism of *Staphylococcus aureus* oleate hydratase (OhyA)

Christopher D. Radka, Justin L. Batte, Matthew W. Frank, Brandon M. Young, and Charles O. Rock

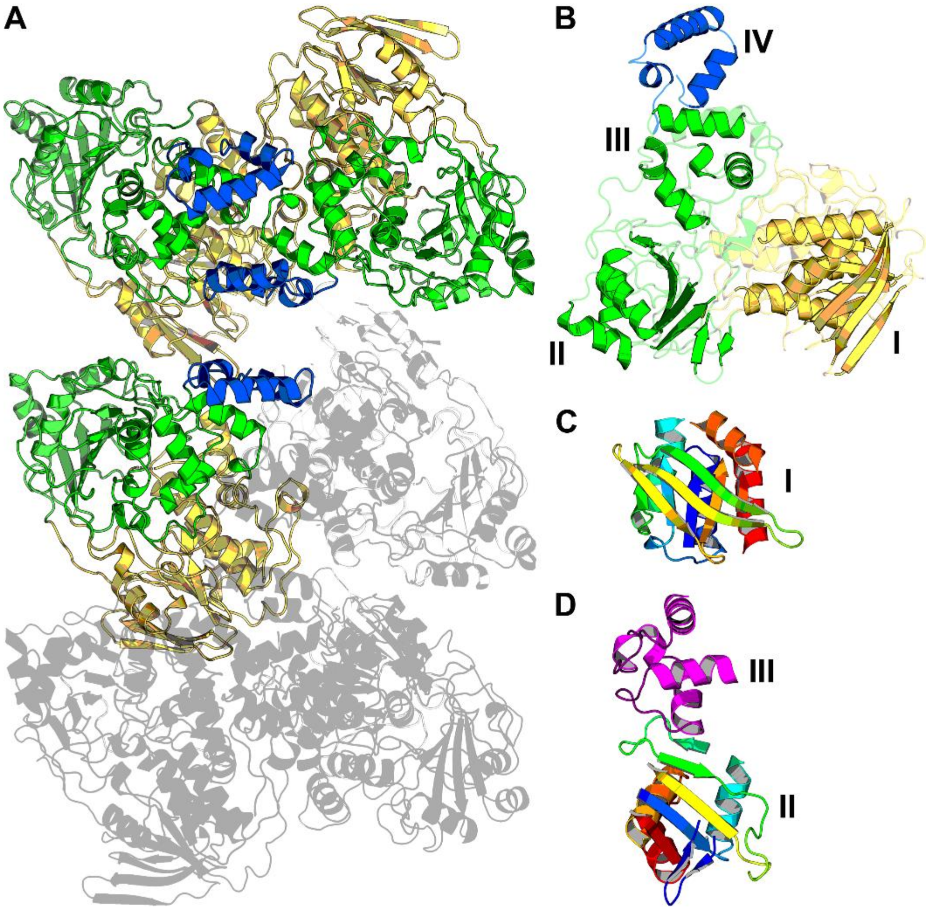

Supplemental Figure S1. Relationship between OhyA structural and functional domains. *A*, Protomers in the OhyA asymmetric unit are colored according to their functional domains: FAD lobe, yellow; fatty acid lobe, green; carboxy terminal domain, blue. Crystallographic symmetry related protomers are shown in gray. *B*, OhyA structural domains (I-IV) defined by Volkov et al. (21) shown in solid colors. Structural domain I corresponds to the FAD functional lobe, structural domains II and III create the fatty acid functional lobe and structural domain IV is the carboxy terminus. *C*, The FAD lobe is formed by structural domain I. The Rossmann fold is colored from blue (amino terminus) to red (carboxy terminus). *D*, The fatty acid lobe is constructed from structural domains II (colored from blue (amino terminus) to red (carboxy terminus) and III (purple) that forms an extended Rossmann-like fold consisting of a five-stranded antiparallel β-sheet flanked by three α-helices on one side, and an α-helical region on the other side.

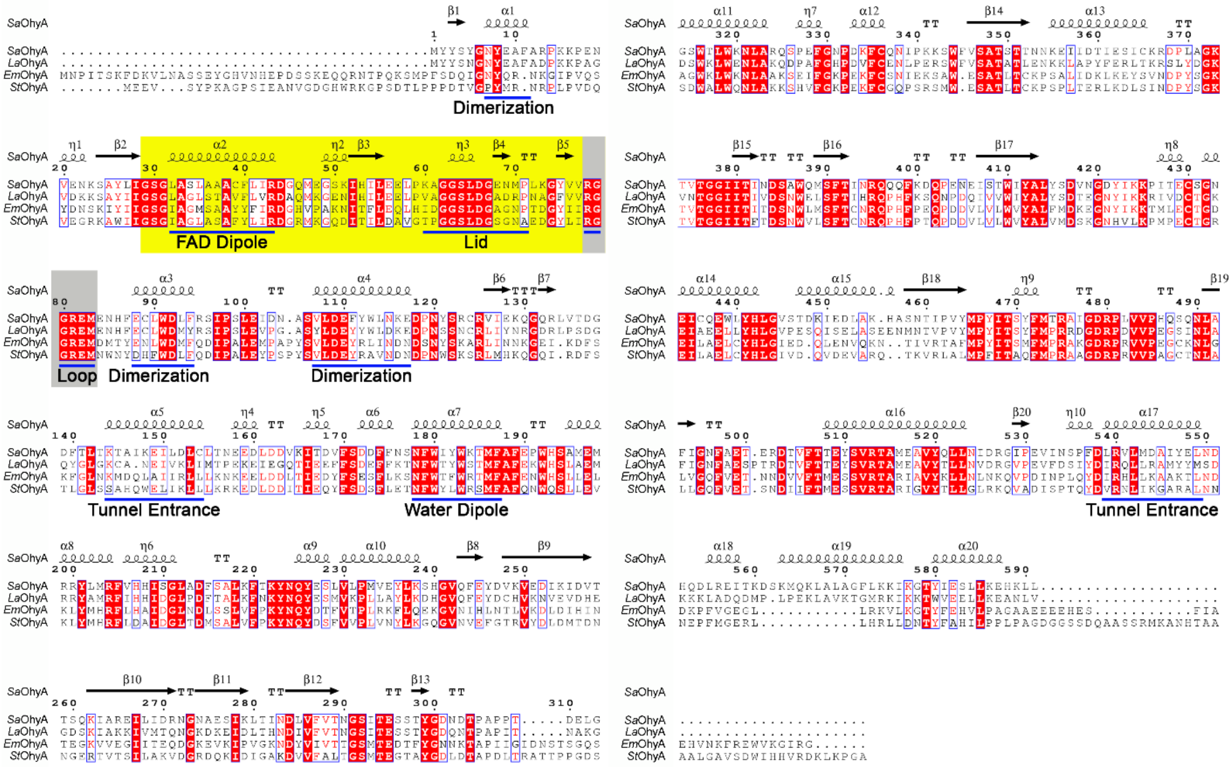

Supplemental Figure S2. OhyA sequence analysis. OhyA Secondary structure mapped onto OhyA ortholog sequence alignment. The *S. aureus* OhyA secondary structural elements are provided above a sequence alignment of *La*OhyA (Uniprot: Q5FL96) *Em*OhyA (Uniprot: C7DLJ6) and *St*OhyA (Uniprot: A0A126NKL7). Functional elements are identified by blue underline below the alignment. Conserved FAD dinucleotide binding motif/phosphate binding signature sequence and catalytic loop indicated with yellow and gray highlight respectively. Primary sequences of oleate hydratases were aligned using Clustal Omega (https://www.ebi.ac.uk/Tools/msa/clustalo/). The *ESPript* program (http://endscript.ibcp.fr/ESPript/ESPript/index.php) was used to visualize sequence conservation and map *Sa*OhyA secondary structure elements onto the multiple sequence alignment. Sequence numbering is assigned to *Sa*OhyA.

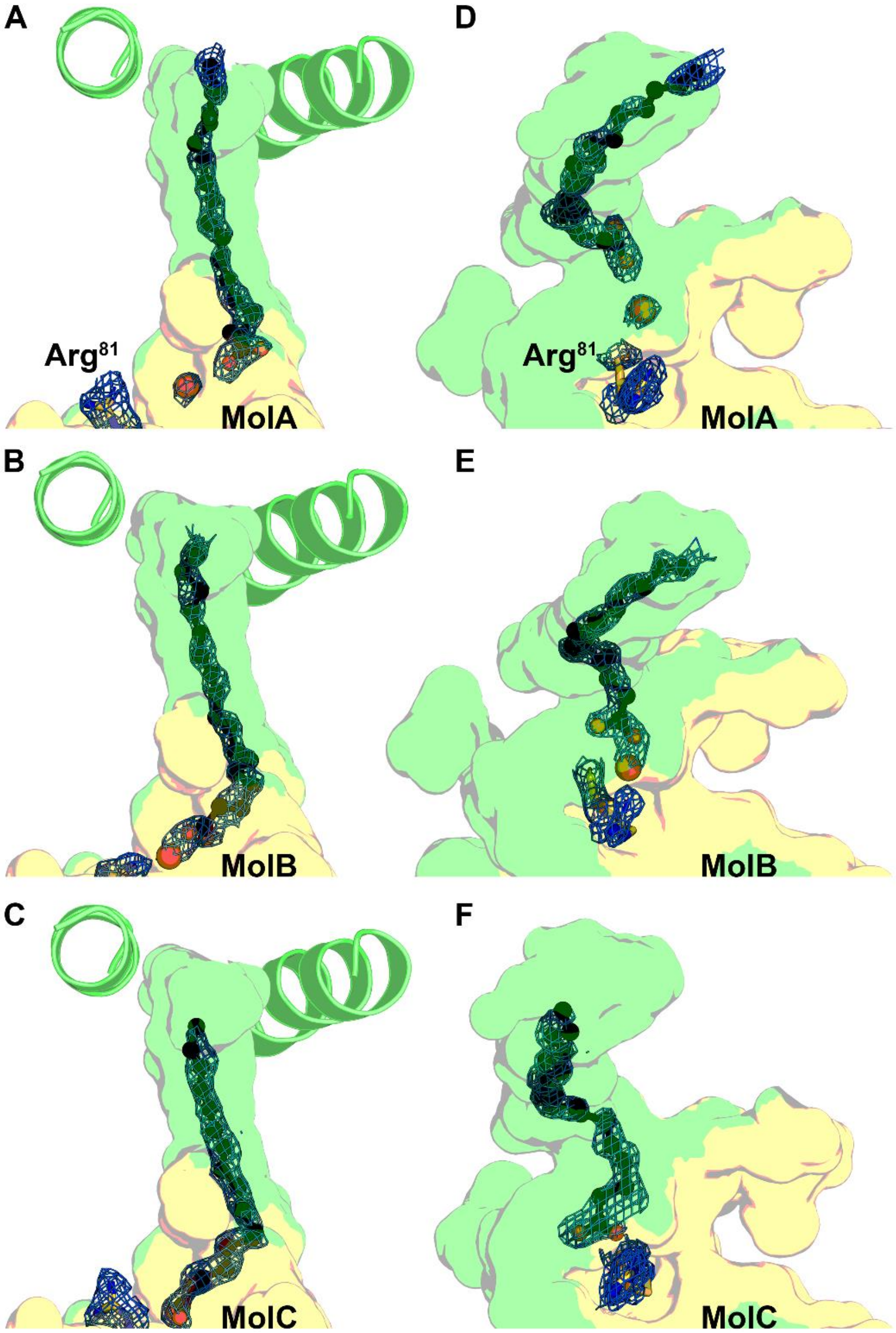

Supplemental Figure S3. Oleate in the hydrophobic tunnel. The carboxylate leads oleate (black) down the hydrophobic substrate binding tunnel to Arg81 in OhyA(E82A)•oleate complex (PDB: 7KAY). The electron density for oleate, Arg81, and the water molecule is calculated from a $2mF_O-DF_C$ map contoured at 1 σ (blue mesh). The FAD lobe is yellow, and the fatty acid lobe is green. *A*, Oleate binds OhyA and is separated from Arg81 by a water molecule (red sphere) in PDB: 7KAY, MolA. *B*, Oleate advances in the tunnel and nears Arg81 in PDB: 7KAY, MolB. Arg81 coordinates oleate through the water molecule. *C*, Oleate directly contacts Arg81 and the water molecule is absent in PDB: 7KAY, MolC. *D*, Rotated view of *A*. *E*, Rotated view of *B*. *F*, Rotated view of *C*.

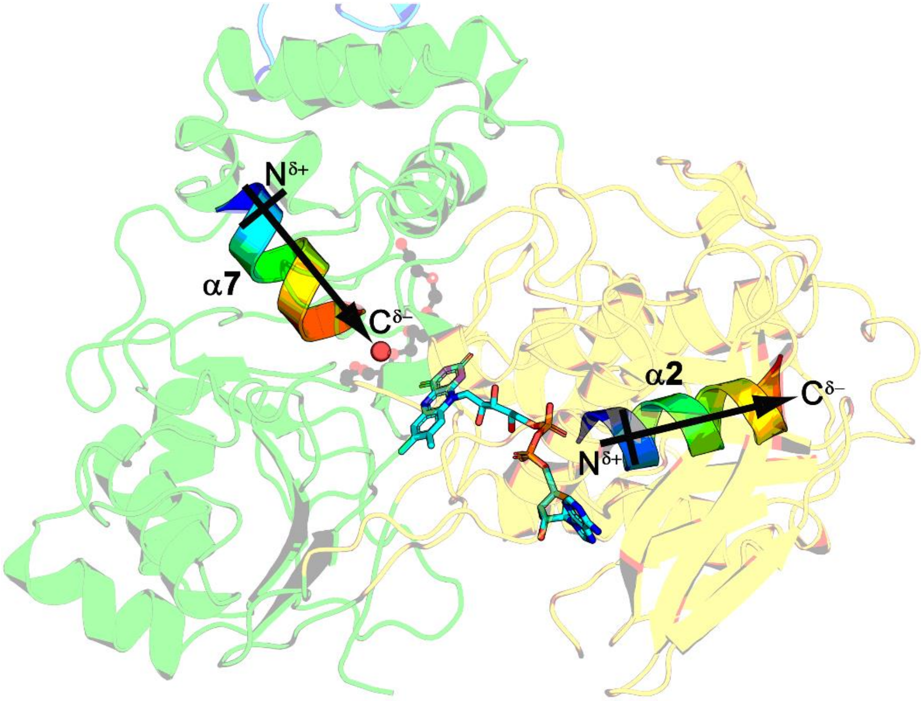

Supplemental Figure S4. OhyA helix dipoles. Orientation of helix dipoles in the OhyA•PEG400•FAD complex (PDB: 7KAW, MolC). The α7 helix dipole stabilizes the active site water substrate and the α2 helix dipole stabilizes the FAD pyrophosphate. The water substrate is the red sphere, and FAD is cyan, the helix dipoles are colored from blue (amino terminus) to red (carboxy terminus), the FAD lobe is yellow, and fatty acid lobe is green.

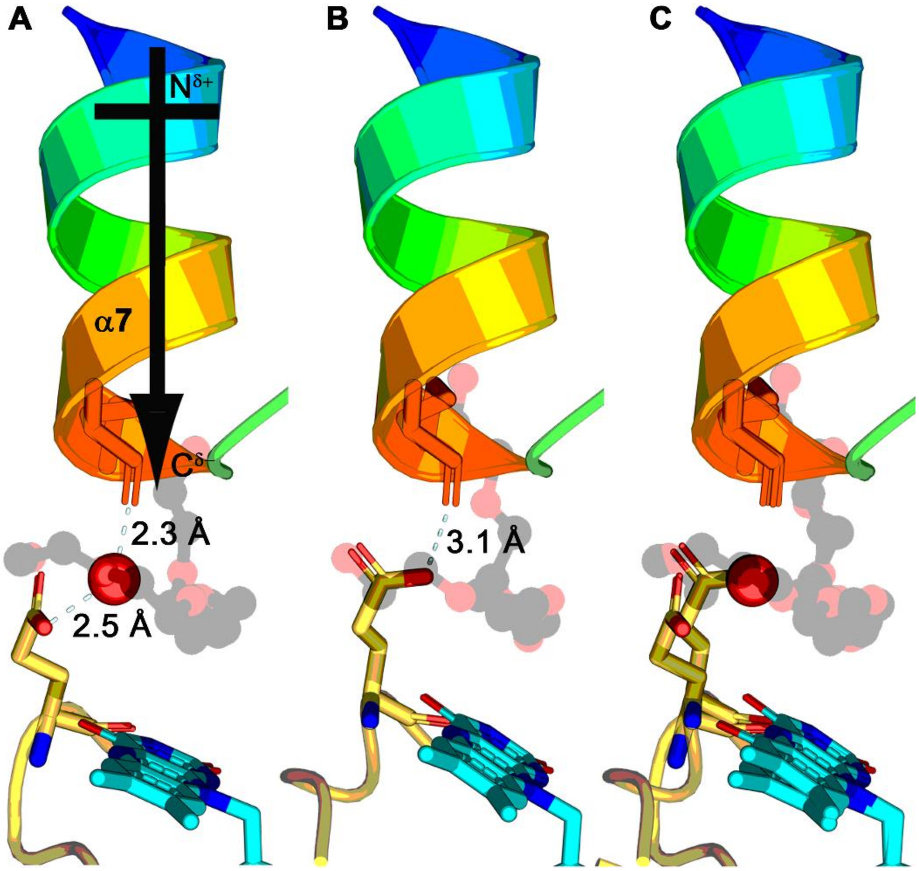

Supplemental Figure S5. The FAD lid compresses the active site. Helix α7 dipole is colored blue (amino terminus) to red (carboxy terminus), FAD is cyan, Glu82 is yellow, water is a red sphere, and PEG400 is black. *A*, The position of the substrate water in OhyA•PEG400•FAD complex with the active site lid in the open conformation (PDB: 7KAW, MolB). *B*, The OhyA•PEG400•FAD complex with the active site lid in the closed conformation (PDB: 7KAW, MolC). The substrate water molecule is absent and Glu82 makes a direct hydrogen bond connection with the backbone carbonyl of Met186. *C*, The overlay of Panels A and B illustrates how the active site compresses after the active site lid is closed.

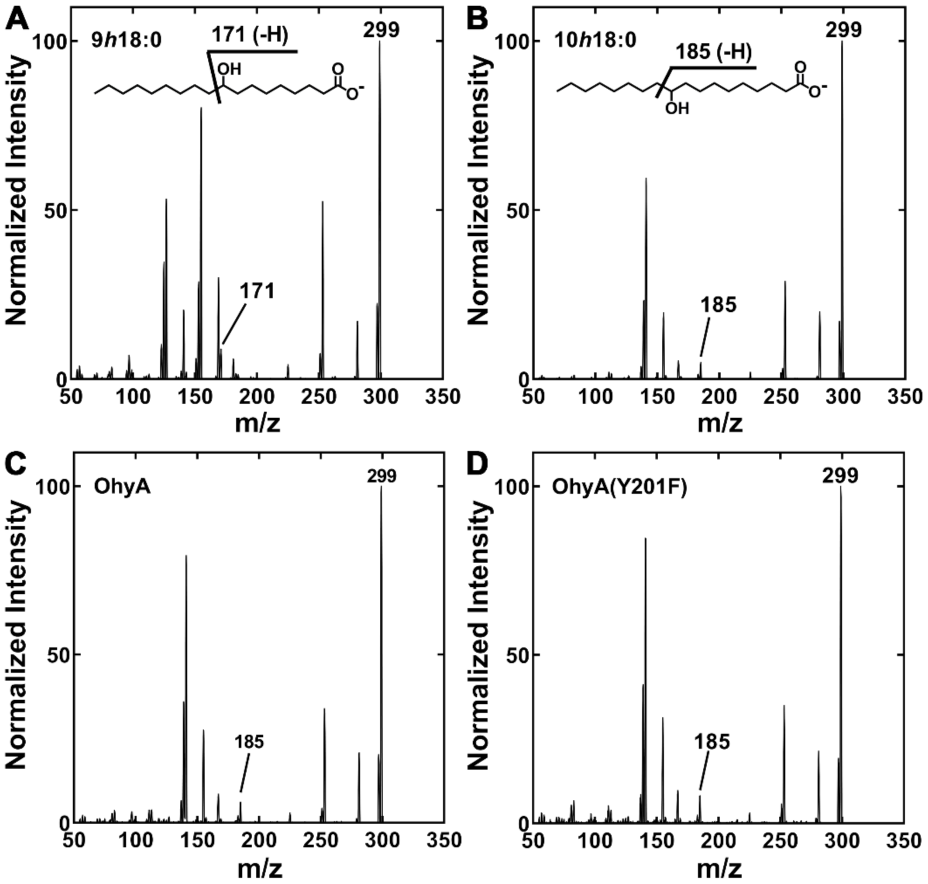

Supplemental Figure S6. Characterization of OhyA and OhyA(Y201F) regioselectivity. Fragmentation of hydroxy fatty acids. The molecular ion *m/z* = 299 corresponds to the hydroxy fatty acid. The fragment *m/z* = 171 is diagnostic for hydroxylation at carbon-9, and the fragment m/z = 185 is diagnostic for hydroxylation at carbon 10 as indicated by the fragmentation diagrams (*insets*). *A*, Mass spectrum of (*R*)-9-hydroxyoctadecanoic acid. *B*, Mass spectrum of *rac*-10-hydroxyoctadecanoic acid. *C*, Mass spectrum of OhyA *h*18:0 reaction product. *D*, Mass spectrum of OhyA(Y201F) *h*18:0 reaction product.

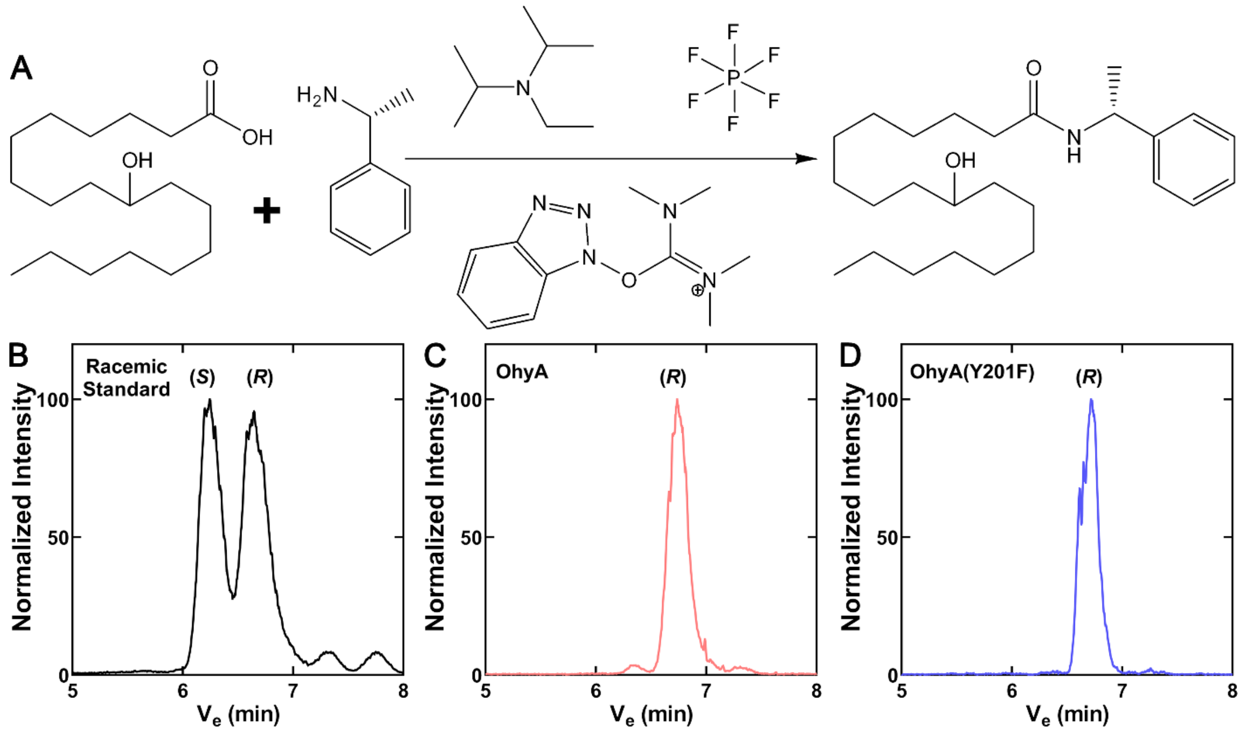

Supplemental Figure S7. Characterization of OhyA and OhyA(Y201F) enantioselectivity. *A*, Reaction scheme depicting (*R*)-α-methylbenzylamine-derivatization method. *B*, Chiral chromatography of (*R*)-α-methylbenzylamine-derivatized *rac*-10-hydroxyoctadecanoic acid. *C*, Chiral chromatography of (*R*)-α-methylbenzylamine-derivatized OhyA *h*18:0 reaction product. *D*, Chiral chromatography of (*R*)-α-methylbenzylamine-derivatized OhyA(Y201F) *h*18:0 reaction product.
